# Comparative population genomic analyses of transporters within the Asgard archaeal superphylum

**DOI:** 10.1371/journal.pone.0247806

**Published:** 2021-03-26

**Authors:** Steven Russum, Katie Jing Kay Lam, Nicholas Alan Wong, Vasu Iddamsetty, Kevin J. Hendargo, Jianing Wang, Aditi Dubey, Yichi Zhang, Arturo Medrano-Soto, Milton H. Saier

**Affiliations:** Division of Biological Sciences, Department of Molecular Biology, University of California at San Diego, La Jolla, CA, United States of America; University of Münster, GERMANY

## Abstract

Upon discovery of the first archaeal species in the 1970s, life has been subdivided into three domains: Eukarya, Archaea, and Bacteria. However, the organization of the three-domain tree of life has been challenged following the discovery of archaeal lineages such as the TACK and Asgard superphyla. The Asgard Superphylum has emerged as the closest archaeal ancestor to eukaryotes, potentially improving our understanding of the evolution of life forms. We characterized the transportomes and their substrates within four metagenome-assembled genomes (MAGs), that is, Odin-, Thor-, Heimdall- and Loki-archaeota as well as the fully sequenced genome of *Candidatus* Prometheoarchaeum syntrophicum strain MK-D1 that belongs to the Loki phylum. Using the Transporter Classification Database (TCDB) as reference, candidate transporters encoded within the proteomes were identified based on sequence similarity, alignment coverage, compatibility of hydropathy profiles, TMS topologies and shared domains. Identified transport systems were compared within the Asgard superphylum as well as within dissimilar eukaryotic, archaeal and bacterial organisms. From these analyses, we infer that Asgard organisms rely mostly on the transport of substrates driven by the proton motive force (*pmf*), the proton electrochemical gradient which then can be used for ATP production and to drive the activities of secondary carriers. The results indicate that Asgard archaea depend heavily on the uptake of organic molecules such as lipid precursors, amino acids and their derivatives, and sugars and their derivatives. Overall, the majority of the transporters identified are more similar to prokaryotic transporters than eukaryotic systems although several instances of the reverse were documented. Taken together, the results support the previous suggestions that the Asgard superphylum includes organisms that are largely mixotrophic and anaerobic but more clearly define their metabolic potential while providing evidence regarding their relatedness to eukaryotes.

## Introduction

Molecular transport systems are essential for all living organisms as sources of nutrition, ions and cofactors as well as for export of toxic substances and end products of metabolism. Our laboratory focuses on the global view of transport, having characterized completely sequenced genomes of various organisms, both prokaryotic and eukaryotic. The recent discovery of the Asgard superphylum has led to the suggestion that these archaea may prove to be closely related to the predecessor of the eukaryotic cell [1]. In view of this potential relationship, we here report analyses of transport systems identified within four metagenome-assembled genomes (MAGs) of these uncultivated archaea as well as one fully sequenced genome, that of *Candidatus* Promethioarchaeum syntrophicum MK-D1 (hereafter referred to as MK-D1) which has been cultured.

It is currently recognized that most of the life on Earth is microbial and remains uncultivated (i.e., not cultured or isolated in a laboratory setting) [1]. These microorganisms are likely to contain an abundance of novel mechanisms that can potentially be applied to improve current efforts in areas such as bioremediation, biotechnology and public health [1, 2]. The Asgard superphylum has emerged as a particularly interesting group of organisms for scientists seeking to elucidate the progression of life from relatively simple prokaryotes to more complex eukaryotes. Phylogenomic analyses, the intersection of studies of genomes and evolution, of the Asgard group have indicated that it may be the closest known archaeal relative to eukaryotes [3, 4].

Microbes occupy almost every niche on Earth and have contributed to the evolution of complex eukaryotic life [5]. Given that most microbes remain uncultivated [1, 6], we anticipate that research on such cells will greatly expand our understanding of life on Earth. The Saier lab has identified transport proteins within species across all domains of life, ranging from bacteria to humans [7]. The characterization of transport proteins has provided a wealth of information regarding all aspects of life [8]. Metagenomics, the study of genomic data derived from environmental samples, provides researchers with the tools to explore the genomic information of uncultivated microbiomes [9–11]. Metagenome-assembled genomes (MAGs), or population genomes (genome bins) are analyzed in this study. In a study such as this one, it should be emphasized that i) the MAGs are incomplete, and consequently, the absence of a transporter does not indicate its absence from the phylum examined; ii) the MAGs may be at least slightly contaminated with DNA fragments from other organisms [12–14], potentially leading to false conclusions regarding the presence of a protein or protein family; iii) a MAG can never provide complete information on a phylum; iv) strain heterogeneity within a MAGs can lead to the appearance of gene duplications that do not actually exist.

In this report, we aim to study the physiological traits of four MAGs and one fully sequenced genome within the Asgard superphylum through the characterization of their transportomes. The transportome of an organism or organismal type conveys information on the metabolic capabilities, substrates acted upon and energy coupling mechanisms that are used to energize transport. Transport proteins constitute about 10% of an average cell’s proteome [15]. Functions include the uptake of nutrients and enzyme cofactors as well as the export of metabolic end products, drugs, and toxins [16, 17]. The powerful field of comparative genomics utilized by bioinformaticians to observe relationships between genomes of different species has contributed to the characterization of consequential transporters, such as those associated with drug resistance in pathogens and oncogenic cells [18, 19]. Additionally, defects in transporters can lead to severe diseases [19], and the characterization of such transporters represents the first step towards the development of targeted treatments.

The Asgard Superphylum, which is phylogenetically related to the TACK archaeal superphylum, is, until very recently (see below), a group of uncultivated Archaea. Prior to the discovery of Asgard, the TACK superphylum was proposed to be the closest archaeal relative to eukaryotes [20]. The number of eukaryotic signature proteins in Asgard archaea surpasses the number found in previously discovered archaeal groups and supports the hypothesis of an archaeal ancestor to eukaryotes [3, 4, 20–22]. The Asgard superphylum contains four phyla named after the Nordic gods: Odinarchaeota (hereafter referred to as Odin), Thorarchaeota (Thor), Heimdallarchaeota (Heimdall) [3, 4, 23] and Lokiarchaeota (Loki). The sampling locations of the extant Asgard reveal that they inhabit many varied environments, but they are particularly prominent in anaerobic methane hydrate seafloor sediments [24].

Phylogenomic analyses of Asgard archaea have challenged the notion that life is separated into three distinct evolutionary lineages or domains: the Eukarya, Bacteria, and Archaea [25]. These studies led to the proposal that an archaeal lineage is the most probable candidate to act as the phagocytosing host cell involved in primary endosymbiotic events. This proposed host cell would have acquired proteobacteria, the precursor to the mitochondrion, resulting in the first eukaryote, in the process termed eukaryogenesis [22, 26]. However, this notion has been challenged, due to research noting that although the Asgard archaea possess the cytoskeletal framework for phagocytosis, they are missing essential pathways necessary for phagocytosis as it occurs in eukaryotes [27].

Eukaryotic signature protein (ESP) homologs in Asgard are involved in cytoskeleton remodeling, vesicle trafficking, and the endomembrane systems in eukaryotes. The functions of ESP homologs, such as actin, GTPase and longin suggests that Asgard organisms contain primitive endolysosome-like capacities and cytoskeletons [3, 4, 28]. Intriguingly, Thorarchaeota encode additional ESPs involved in intracellular trafficking pathways such as Endoplasmic Reticulum-to-Golgi transport, secretion and autophagy [4, 28]. These eukaryotic-like proteins in Thor, include some components of the TRAPP complex, which is a highly conserved multi-subunit complex conserved from yeast to humans [29], and Sec23/Sec24 protein families, which are essential domains in vesicular transport [30].

Most Asgard organisms are probably anaerobic and mixotrophic, and these suggestions are supported here. Thus, the Asgard archaea have been shown to use a mixture of energy and carbon sources from different trophic modes such as heterotrophy and autotrophy [24, 31]. The Wood-Ljungdahl carbon fixation pathway (WLP) [or acetyl-coenzyme A (CoA) pathway], hypothesized to be the oldest carbon fixation pathway on Earth, represents a means for the synthesis of acetyl-CoA from two carbon dioxide molecules concomitant with the generation of energy [32]. All Asgard proteomes contain key enzymes of the WLP and therefore may be capable of acetogenesis [23, 31, 33]. Furthermore, these organisms may use the end products of WLP as electron acceptors and therefore may be capable of heterotrophic growth [23]. Studies have revealed the presence of essential proteins involved in the import and degradation of organic molecules such as carbohydrates and proteins [23, 24, 33–35]. It should be noted that the Asgard proteomes contain the essential components of glycolysis except for the key initial enzyme, hexokinase [24]. Thorarchaeota may play a role in reducing intermediate sulfur compounds generated by the oxidation of sulfides in the sulfate-methane transition zones of its sediment sampling location [23]. In contrast to the anaerobic Lokiarchaeota and Thorarchaeota, recent research has shown that some Heimdallarchaeota MAGs encode oxygen-dependent metabolic pathways [35].

Although the Asgard archaeal relationship to eukaryotes is still disputed [12], the findings reported above have been interpreted to support a two-domain tree of life, wherein the eukaryotes are considered a monophyletic group stemming from the domain Archaea [3, 4, 22]. We chose to contribute to this discussion at the level of transport systems by studying the relationships that Asgard transporters share with eukaryotes, other archaea, and bacteria. Although more recent research has annotated the MAGs of the Asgard superphylum for the purpose of characterizing their physiology [36], there has been little effort to compare the presence of proteins responsible for membrane transport between the Asgard phyla although Bulzu et al. [35] have characterized the metabolic capabilities and transporters in the Heimdallarchaeota MAG.

To contribute to the knowledge of the overall physiology of Asgard, we have sought to identify the types of transporters encoded within the four MAGs and MK-D1, as well as their probable substrates and concomitant physiological [37]. We here report that the five Asgard transportomes analyzed provide evidence supporting Asgard’s closer phylogenomic relationship to eukaryotes but also show that a majority of transport proteins are more similar to those of prokaryotes than to those of eukaryotes. We also show that *pmf*-driven secondary carriers outnumber ATP-drive primary active transporters, presumably a reflection of their energy-generating mechanisms.

Towards the end of our Asgard MAG studies, an Asgard archaeon was cultivated in the laboratory together with two “helper” organisms that provided nutrients and other essential compounds, allowing slow growth [38]. This Asgard organism, designated ’*Candidatus* Prometheoarchaeum syntrophicum strain MK-D1’, is an anaerobic, extremely slow-growing (division time of about 20 days), small coccus (around 550 nm in diameter) that degrades amino acids and peptides through syntrophy with co-cultured *Methanogenium* (archaeon) and *Halodesulfovibrio* (bacterium) partners, reaching a final cell density of only about 10^5^ cells per ml. These dissimilar organisms may catalyze interspecies hydrogen and/or formate transfer. Eventually, *Halodesulfovibrio* was eliminated without preventing growth altogether. The complete genome of MK-D1 was sequenced and made available [38]. We report analyses of transport proteins encoded within this genome and compare them with the Asgard MAGs.

## Methods

### Identification of single-component transport systems

The protein sequences of the four Asgard MAGs, those of Odin, Thor, Heimdall and Loki, as well as the MK-D1 full genome, were extracted from the corresponding assemblies in NCBI. One proteome from each phylum was chosen for analyses based on the total length of their sequenced MAGs, the level of completeness, the number of citations associated with the MAGs and their relevance to the evolution of eukaryotic complexity. Three chosen MAGs were the largest of their respective phyla and had been cited to emphasize Asgard’s close phylogenomic relationship to eukaryotes. For Thor, we chose the second largest metagenome because it was instrumental in the grouping of Thor into the Asgard superphylum [4, 39, 40]. Furthermore, the largest Thor MAG that we did not analyze (previously described as a part of bathyarchaeota [41]) had already been rigorously characterized [23, 41, 42] and we sought to contribute to an understanding of the lesser annotated Thor MAG.

The proteomes of each of the four Asgard phyla were screened against all of the proteins contained in the Transporter Classification Database (www.tcdb.org) for homologues using GBLAST [43]. GBLAST runs BLAST [44] to compare protein sequences from the proteomes to proteins within TCDB. GBLAST reports E-values, protein lengths, sequence identities, alignment coverage, hydropathy profiles estimated with the Web-based Hydropathy, Amphipathicity and Topology (WHAT) program [45], Transmembrane segments (TMSs) inferred with HMMTOP [46], and the substrate(s) associated with the matching TC system.

Candidate homologues were identified if alignments showed E-values < 10^−5^ with alignment coverages ≥ 60% when proteins were of comparable size. Lower coverages were also allowed when one of the proteins was > 60% larger than the other to identify potential protein fusions. Since two proteins may display a significant alignment score due to aligned hydrophilic regions, it was necessary to examine the alignments to prevent false positives in assignments where there is no similarity in the transmembrane domain [38, 47].

Confidence in homology inferences was strengthened when hydropathy alignments showed overlapping TMSs and conserved Pfam [48] domains (domains are conserved parts of a protein that can fold and function independently). Putative orthologous proteins were identified across all MAGs and MK-D1 when high-confidence and high-coverage alignments (allowing for potential fusions) with the homologous protein in TCDB were found.

Proteins with no BlastP hits or showing poor scoring alignments (E-values ≥ 10^−5^) when compared to TCDB, were also examined to identify distant members of established TC families and novel families of transporters. The main goal in this case was to increase the scope of transporter sequence diversity represented in TCDB. Distant members of a given family were identified when candidate transporters in the proteomes (having at least 4 TMSs) shared the same Pfam domains or clans as established members of the family in TCDB. If no Pfam domains were found in candidate transporters, we attempted to project the characteristic domain(s) of the target TC family onto the candidate transporter using a method previously published [49, 50].

### Identification of multicomponent transport systems

**Fig 1:**
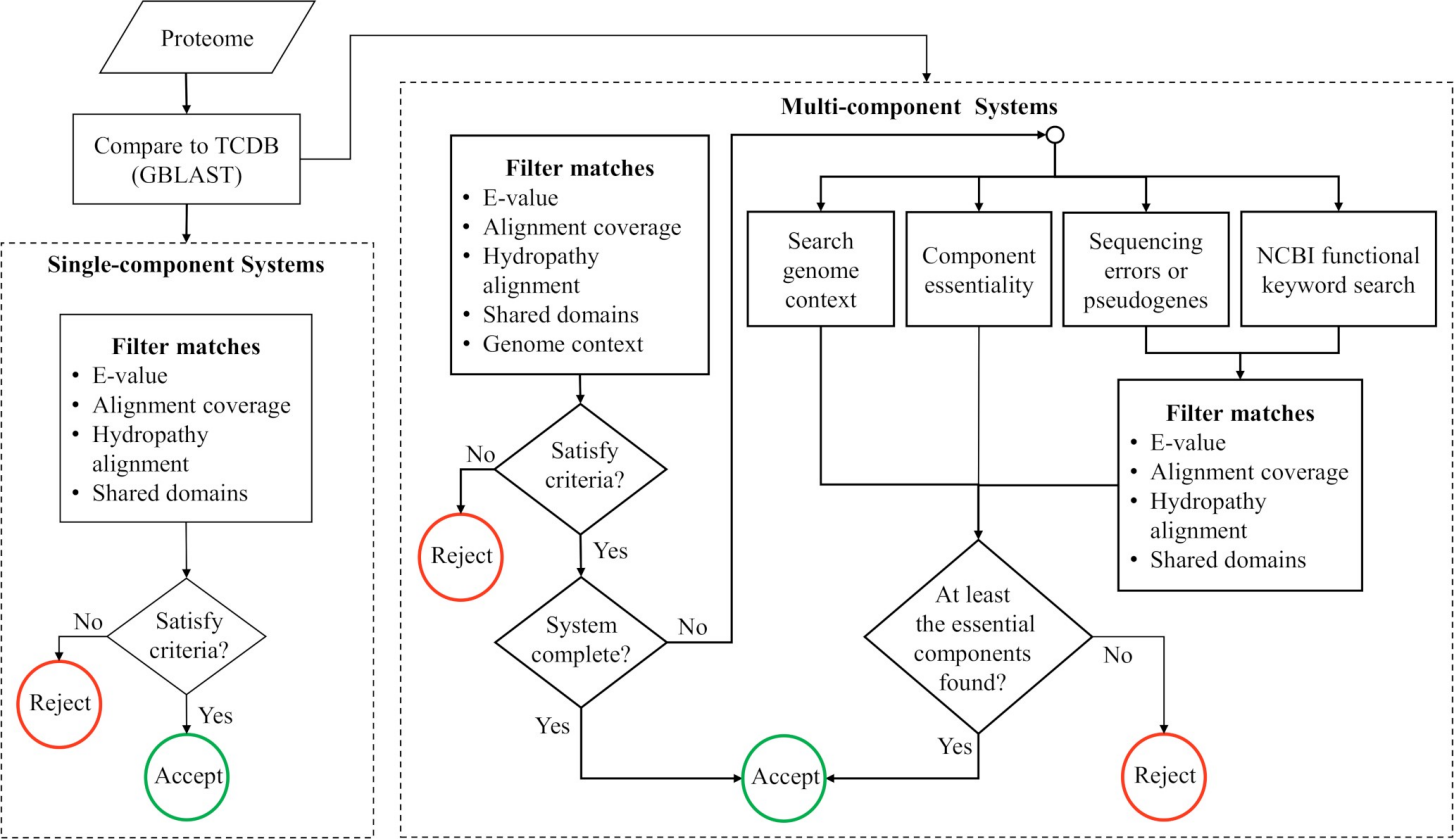
Strategy for characterizing transport systems. To characterize the five Asgard transportomes, candidate single- and multi-component systems were identified in each proteome following the multiple criteria shown in this flow chart. See methods for a detailed description.

In addition to the strategies listed above, multicomponent transport systems were identified by further taking into consideration the genomic context of the components (in prokaryotes, multicomponent systems are often organized in operons), comparisons against transporters with the expected annotation in UniProt/NCBI, the essentiality of the components (missing components are tolerated when they are non-essential for transport function), and the possibility that different transport systems may share components (e.g., ATPases, some receptors, and other accessory proteins). When no matches in TCDB were found for a specific component, we queried UniProt/NCBI for all proteins with the annotation of the missing component. These proteins were then collected and BLASTed against the corresponding (meta)proteome. If at this point, an essential component could not be found in the genome, we searched for the footprint of the gene at the DNA level with BlastX, since it may have not been annotated in the assembly because a) the sequence is not yet complete, b) there are sequencing errors, or c) the protein-encoding gene became a pseudogene [51]. A flowchart presenting the strategy used to identify homology between protein sequences from the Asgard MAGs and MK-D1 to annotated systems in TCDB is illustrated in Fig 1.

## Results and discussion

Asgard genomic material is represented by the MAGs and one full genome sampling the four recognized phyla, which consist of segments of overlapping DNA (i.e., contigs or scaffolds). The MAGs of Loki and Heimdall analyzed here contained over 5 million base pairs each while those of Thor and Odin contained roughly 3 million and 1.5 million base pairs, respectively [3, 4]. The completely sequenced genome of *Candidatus* Promethioarchaeum syntrophicum MK-D1, within the Loki phylum, contains 4.43 million base pairs. Further information on the selected Asgard MAGs and MK-D1 that we examine here is provided in Table 1.

**Table 1:** Properties of four selected MAGs and one fully sequenced genome from the Asgard phyla.

	MAG/Genome				
	Odin^‡^	Thor^†^	Heimdall*	Loki^Ψ^	MK-D1^#^
Assembly accession	ASM194066v1	ASM194070v1	ASM194064v1	ASM98684v1	ASM800077v1
Total proteins	1584	2914	5514	5384	3916
Total transport proteins	164 (10.3%)	291 (10%)	416 (7.5%)	325 (6%)	363 (9.3%)
Size (Mbp)	1.46	2.86	5.68	5.14	4.43
Contig count	9	264	157	504	Complete genome
Sampling location	Lower Culex Basin hot spring	Marine sediment Aarhus Bay, Baltic Sea	Loki’s castle hydrothermal vent sediment	Loki’s castle hydrothermal vent sediment	Deep marine sediment

“Total proteins” indicates the numbers of proteins annotated in the corresponding assemblies as of 2020. “Total transport proteins” indicates the number of proteins that were inferred in this study to be homologous to a TCDB transporter, and percentages relative to total proteins are shown in parenthesis. “Contig count” refers to the number of contigs provided in the assembly.

^‡^ *Candidatus* Odinarchaeota archaeon LCB_4.

^†^ *Candidatus* Thorarchaeota archaeon AB_25.

* Candidatus Heimdallarchaeota archaeon LC_3.

^Ψ^ *Lokiarchaeum sp*. GC14_75.

^#^ *Candidatus* Prometheoarchaeum syntrophicum MK-D1.

### Transport types in four Asgard MAGs and one fully sequenced genome

The Transporter Classification (TC) system classifies transport systems using a five-tier system based on their structures and functions. The first tier is divided into 5 well-defined categories (TC classes 1–5) and 2 less well-defined categories (TC classes 8–9). The five well defined classes are designated: 1) channels/pores, 2) electrochemical potential-driven transporters known as secondary carriers, 3) primary active transporters, 4) group translocators, and 5) transmembrane electron carriers. The 2 less well-defined classes are 8) auxiliary transport proteins and 9) putative or incompletely characterized transport systems. Following the TCDB convention, transporters are further ranked in increasingly specific groups: subclass, family, subfamily, and lastly, the actual transport system [17].

As outlined in Methods, the five Asgard proteomes were screened using GBLAST to identify candidate transporters. Of importance to note is that since we are analyzing 4 MAGs, we can only identify what transport systems are present, and proteins that are regarded as not found in a particular MAG do not reflect proteins missing from the actual set of organisms because the genomic data are incomplete. Our results revealed that Heimdall contains the most identified transport proteins (416) and Odin contained the least (164), corresponding roughly to the amount of sequenced DNA available for analysis (Table 1). The remaining Asgard proteomes, those of Thor, Loki and MK-D1 contain 291, 325 and 363 total transport proteins, respectively. Loki contains the smallest percentage of transport proteins (6%) compared to the other Asgard transportomes while Odin, despite containing the smallest MAG size, encodes the largest percentage of transport proteins (10.3%) (Table 1). S1 Table shows the top blast matches between Asgard proteins and TCDB. In addition, the table indicates the inferred substrates for each system.

TC subclass 1.A consists of α-type channels. These are ubiquitously found throughout all domains of life. They usually catalyze the transport of substrates through a pore or channel within a membrane by an energy-independent process. The five Asgard transportomes were found to include 6–25 α-type channels. MK-D1 contains 25 α-type channels, more than double that for Odin, Thor and Loki (Table 2).

**Table 2:** Numbers of identified Asgard transport systems per TC subclass.

Subclass	Odin	Thor	Heimdall	Loki	Totals	MK-D1
					(4 MAGs)	
1.A	6 (6)	4 (4)	16 (16)	8 (8)	34 (34)	25 (25)
Alpha-type channel						
1.C	0 (0)	0 (0)	0 (0)	2 (2)	2 (2)	2 (2)
Pore-forming toxins						
1.E	0 (0)	1 (1)	3 (3)	0 (0)	4 (4)	0 (0)
Holins						
2.A	36 (38)	62 (62)	76 (79)	85 (85)	259 (264)	106 (106)
Porters (uniporters, symporters, antiporters)						
3.A	22 (62)	40 (110)	64 (169)	44 (91)	170 (432)	45 (106)
P-P-bond-hydrolysis-driven transporters						
3.D	4 (28)	7 (37)	6 (31)	5 (24)	22 (120)	1 (7)
Oxidoreduction-driven transporters						
3.E	0 (0)	0 (0)	0 (0)	0 (0)	0 (0)	1 (1)
Light absorption-driven transporters						
4.C	1 (1)	9 (9)	6 (6)	25 (25)	41 (41)	14 (14)
Acyl-CoA ligase-coupled transporters						
4.D	2 (2)	2 (2)	9 (9)	3 (3)	16 (16)	2 (2)
Polysaccharide synthase/exporters						
4.F	1 (1)	2 (2)	4 (4)	6 (6)	13 (13)	5 (5)
Choline/Ethanolamine Phosphotransferase						
5.A	0 (0)	1 (1)	7 (15)	2 (2)	10 (18)	1 (1)
Transmembrane 2-electron transfer carriers						
8.A	2 (2)	3 (4)	7 (7)	3 (3)	15 (16)	8 (8)
Auxiliary transport proteins						
9.A	1 (1)	3 (4)	3 (3)	1 (1)	8 (9)	2 (3)
Transporters of unknown biochemical mechanisms						
9.B	23 (23)	55 (55)	74 (74)	75 (75)	227 (227)	83 (83)
Putative transport proteins						
**Totals**	98 (164)	189 (291)	275 (416)	259 (325)	821 (1196)	295 (363)

Values in parentheses represent the total number of proteins per subclass. Column 6 (Totals) presents total systems and proteins on the preceding 4 MAGs in columns 2–5.

TC subclass 1.B includes outer membrane porins, which usually, but not always, form beta barrel structures, especially in Gram-negative bacteria. However, those in actinobacteria and eukaryotic organelles may be of alpha structure. Not even one outer membrane porin homolog was identified in the Asgard proteomes, supporting the earlier suggestion that the Asgard organisms are monoderms with a single cytoplasmic membrane, lacking an outer membrane [38].

TC subclass 1.C consists of pore-forming toxins. These systems are synthesized in one cell, secreted, and form transmembrane pores in another cell which leads to lysis of the latter cell. Loki and MK-D1 were the only Asgard proteomes in which we identified proteins belonging to this subclass. Two homologs of the bacterial hemolysin A (B-Hemolysin A) family (TC# 1.C.109) were found in Loki. Loki B-Hemolysin A homologs presumably form pores in membranes of other cells and consequently cause cell lysis [52], a potential source of nutrition for Loki. MK-D1 has one homolog of the pore-forming hemolysin III from the Hly III family (TC# 1.C.113).

TC subclass 1.E consists of holins. Holins perform a variety of functions in bacteria, but mainly serve to promote cell death [53, 54]. They are encoded by bacteria and bacteriophages. In both cases, they induce cell death by forming pores in the cytosolic membrane of the bacteria that produce them, and thereby, release endolysins that hydrolyze the cell wall to cause cell death [53]. Holin homologs were identified in Thor and Heimdall, but not in the other proteomes (Table 2). These putative holin homologs, of unknown function, all belong to the Putative 3–4 TMS Transglycosylase-associated Holin (T-A Hol) family (TC# 1.E.43).

TC subclass 2.A consists of porters. Porters (e.g., antiporters, symporters, and uniporters) are secondary carriers that permit transport in a carrier-mediated process that in prokaryotes is usually driven by an electrochemical potential. Porter systems make up the largest known transporter type in all Asgard proteomes, constituting on average 32.8% of the total transport systems, performing a diversity of functions.

TC subclass 3.A consists of primary active transporters that derive their energy for transport from the hydrolysis of diphosphate bonds. Based on the total number of proteins involved in transport, this group is the most abundant transport protein type for all Asgard proteomes. However, most primary active transporters are multicomponent systems, and they make up the second largest known transporter type. Odin encodes 22 of these systems while the other Asgard proteomes contain on average about 49 (Table 2).

TC subclass 3.D includes the oxidoreduction-driven transporters. These systems derive energy for transport of an ion, H^+^ or Na^+^, from the transfer of electrons from a reduced substrate to an oxidized substrate. Oxidoreduction-driven transport systems were identified in all proteomes (Table 2).

TC class 4 consists of group translocators that either loosely or tightly couple substrate modification to the transport process. TC subclass 4.C represents the Acyl CoA ligase-coupled transporters and is well represented in all transportomes, except that of Odin where only one homolog was found (Table 2). TC subclass 4.D includes the polysaccharide synthase/exporters, and all transportomes contain at least 2 homologs. TC subclass 4.F consists of the choline/ethanolamine phosphotransferase 1 and is represented in all proteomes. Similarly to subclass 4.D, all proteomes have at least two homologs, except for that of Odin which has only one.

TC class 5 represents the transmembrane electron carriers. This class influences the membrane potential by transporting either one or two electrons across the membrane in either direction, from the inside of the cell to the outside, or vice versa. This class is divided into two subclasses depending on the number of electrons transported across the membrane in a single step; TC subclass 5.A transports two electrons as a pair, while 5.B transports single electrons across the membrane. While no transmembrane electron carriers of any kind were identified in Odin, two-electron transmembrane carriers are represented in the remaining Asgard transportomes. Heimdall contains 7 systems of two-electron transmembrane carriers compared to Thor, Loki and MK-D1 were we found on average only one such system (Table 2).

TC subclass 8.A consists of auxiliary transport proteins. These proteins do not participate directly in transport, but in some way influence or facilitate transport and do not belong to another TC multicomponent system. Systems in this subclass were identified in all Asgard transportomes (Table 2).

TC subclass 9.A contains established transporters of unknown biochemical mechanism. These transporters’ functions are known, but no described mode of transport or energy coupling mechanism has been reported. Each of the Asgard transportomes contains at least 1 system from TC subclass 9.A. TC subclass 9.B is comprised of putative uncharacterized transport proteins. This subclass is the second-best represented group of transport systems of the Asgard transportomes. An overview of the presence of transport systems in the five Asgard transportomes can be found in Table 2.

### α-Type channel proteins (TC subclass 1.A)

The ubiquitous Voltage-gated Ion Channel (VIC) superfamily (TC# 1.A.1) [55] was represented in Heimdall and MK-D1. Heimdall has one protein homologous to the VIC transport system 1.A.1.13.8, which is predicted to be capable of transporting potassium at low membrane potentials [56]. The eukaryotic-like protein in MK-D1 homologous to system 1.A.1.31.1 is uncharacterized.

The five Asgard proteomes have multiple homologs within different families involved in regulating calcium ion levels across lipid bilayers. Each of the Asgard proteomes contains 1–3 proteins homologous to transporters within the Calcium Load-activation Calcium Channel (CLAC) Family (TC# 1.A.106). These Asgard proteins display reasonable levels of sequence similarity (E-value < 10^−15^) and exhibit over 90% coverage to members of the TC subfamily 1.A.106.2, which is exclusively made up of archaeal proteins. We identified in all Asgard proteomes, with the exception of Thor, homologs of the Presenilin ER Ca^2+^ Leak Channel (Presenilin) Family (TC# 1.A.54). Heimdall, Loki and MK-D1 each contain one protein homologous to characterized eukaryotic presenilins, while Odin and Heimdall have homologs of presenilins that have been biochemically shown to act both as proteolytic enzymes and cation channels in archaea [57]. In all four Asgard phyla, these channels may function in controlling the influx of calcium ions into the cell [58].

Heimdall, Thor and Loki each contain 1–3 homologs of the Small Conductance Mechanosensitive Ion Channel (MscS) Family (TC# 1.A.23). These proteins relieve osmotic pressure in hypotonic solutions through the release of ions in response to cellular expansion [59, 60]. Interestingly, we were able to identify only in MK-D1 a homolog of the Large Mechanosensitive Ion Channel (MscL) family (TC# 1.A.22).

All five Asgard proteomes contain homologs of the Cation Channel-forming Heat Shock Protein-70 (Hsp70) family (TC# 1.A.33). Hsp70 has been identified across all domains of life, and in eukaryotes, they are capable of forming membrane channels [61, 62]. However, no evidence for this characteristic has been shown in prokaryotes. All Asgard metagenomes have 1–3 homologs of the chaperone protein DnaK (TC# 1.A.33.1.4). The significantly high sequence similarities (E-value < 10^−90^) and coverages (> 95%) between these Asgard proteins and the TCDB systems are clearly indicative of their homologous nature.

In agreement with previous research [4], we identified homologs of the eukaryotic-specific Magnesium Transporter1 (MagT1) family (TC# 1.A.76) in all Asgard proteomes. All MagT1 homologs in these proteomes belong to subfamily 1.A.76.2, whose members are part of the oligosacharyltransferase complex responsible for the transfer of an oligosaccharide chain onto asparagine residues [63, 64]. It is possible that these proteins also transport Mg^2+^. In addition to MagT1, a homolog the eukaryotic subfamily 1.A.26.2 within the Mg^2+^ Transporter-E (MgtE) family (TC# 1.A.26), involved in the uptake of Mg^2+^ into vertebrate cells [65], was identified in Heimdall. Except for Loki, we were able to identify other types of prokaryotic magnesium ion transporters in all transportomes. Such transporters include a putative system from the Cylcin M Mg^2+^ Exporter (CNNM) family (TC# 1.A.112) in Heimdall, a homolog of a bacterial putative Mg^2+^-ATPase (TC# 3.A.3.4.2) in Odin, a homolog of the CorA Metal Ion Transporter (MIT) family (TC# 1.A.35) in both Odin and MK-D1, and a homolog of the putative Mg^2+^ Transporter-C (MgtC) family (TC# 9.B.20) in Thor and MK-D1.

Other channels were found in individual Asgard proteomes. Heimdall contains a homolog of the Calcium Transporter A (CaTA) family (TC# 1.A.14). Odin contains a single putative homolog of the Fluoride Channel (Fluc) family (TC# 1.A.43). MK-D1 has multiple paralogs of the Pore-forming NADPH-dependent 1-Acyldihydroxyacetone Phosphate Reductase (Ayr1) family (TC# 1.A.115), which include proteins most similar to eukaryotic (TC# 1.A.115.1.1), archaeal (TC# 1.A.115.1.2) and bacterial (TC# 1.A.115.1.3 and 1.A.115.1.5) systems. In summary, we found in MK-D1 and Heimdall the largest numbers of alpha-type channel systems (25 and 16, respectively), which are on average more than twice the numbers of alpha-type channels identified in Odin, Thor and Loki (Table 2). Most of these channels seem to be unknown, nonspecific for ions, or specific for cations such as calcium and magnesium. The presence of eukaryotic-like calcium and magnesium channels in these transportomes provides some evidence supporting Asgard’s phylogenomic relationship to eukaryotes. While the eukaryotic calcium channels have been reported to function in calcium ion level regulation between endo-membranes in eukaryotes [58], it is possible that the function of these transport systems in Asgard may be to transport calcium ions across the plasma membrane rather than the endomembrane. Taken together, the calcium channels in Asgard, which are homologous to characterized and putative calcium channels, may play a role in calcium signaling or the regulation of calcium influx into the cell.

### Secondary carriers (TC subclass 2.A)

In terms of transport systems per proteome, secondary carriers make up the largest fraction of the transportome. We found that 27.6–36.7% of the transport systems in the Asgard transportomes belong to this subclass, with Loki and MK-D1 having the most (Table 2). The largest family of homologous porters present in nature, the Major Facilitator Superfamily (MFS), is the best represented group of secondary carriers in Asgard [47]. The MFS is a large and diverse group of uniporters, symporters, and antiporters [66]. The MFS is currently composed of 89 families within 2.A.1 and 15 families outside of 2.A.1, but only homologs of families within 2.A.1 and/or 2.A.2 were identified in all proteomes. Of the five Asgard transportomes, MFS transport systems make up the smallest fraction in Odin (7.1%) and the largest in Loki (14.3%) and MK-D1 (20.7%). In Thor and Heimdall, they represent 12.2% and 11.3% of their transportomes, respectively. The primary role of MFS transporters in Asgard appears to be multi drug resistance. Of the MFS porters, the most common type in all Asgard phyla is the Drug:H^+^ Antiporter-1 (DHA1) Family (TC# 2.A.1.2). Although there is extensive evidence regarding the capacity of DHA1 family members to catalyze drug efflux in all domains of life [66, 67], it cannot be assumed that drug efflux is the primary physiological function of these transporters. Homologs from the Drug:H^+^ Antiporter-2 (DHA2) Family (TC# 2.A.1.3) were identified in Thor and Heimdall, including suggested riboflavin and siderophore exporters [68, 69]. Indeed, numerous homologs of the Drug:H^+^ Antiporter-3 (DHA3) Family (TC# 2.A.1.21) were detected in all Asgard transportomes except that of Odin. These DHA3 homologs have been shown to confer macrolide (a type of microbial antibiotic) resistance in *Streptococcus pneumoniae* [70, 71]. DHA homologs catalyze the transport of molecules by hydrogen symport or antiport. Therefore, a hydrogen gradient may be essential in all Asgard phyla to sustain the transport of toxins and thus, promote drug-resistance phenotypes.

Other MFS homologs include pyruvate/H^+^ symporters (TC# 2.A.1.11.3) that were detected in Odin, Thor, Loki, and MK-D1. This system in *E*. *coli* is known to take up pyruvate [72]. Homologs of the Putative Aromatic Compound/Drug Exporter (ACDE) family (TC# 2.A.1.32) were found in all Asgard transportomes except that of Thor. A member of the ACDE family (TC# 2.A.1.32.1) was found in Loki, while Odin, Heimdall, and MK-D1 encode a putative corynebactin (siderophore) exporter (TC# 2.A.1.32.2) [73]. Thor encodes a member of the Enterobactin Exporter (EntS) family (TC# 2.A.1.38), whose homolog in *Salmonella* is known to export the siderophore, enterobactin [74]. At least one member of families capable of siderophore export (ACDE, EntS, or DHA2) was found in all Asgard proteomes. Notably, we cannot confidently claim that the putative siderophore exporters within these Asgard metagenomes function to sequester iron (Fe^3+^) for eventual uptake by separate siderophore uptake systems for the following reasons. Firstly, siderophores chelate ferric iron, not ferrous iron, and the latter is most likely to be present in the anaerobic environment from which these Asgard metagenomes were sequenced [75]. Secondly, the putative siderophore exporters are also homologous to multidrug resistance proteins, and drug export may be their physiological function in Asgard. Lastly, iron-siderophore uptake systems were not found in any of the five Asgard proteomes, although as noted above, the absence of a transporter encoded within a particular MAG does not establish its absence.

Heimdall and MK-D1 contain a homolog of the Organophosphate:P_i_ Antiporter (OPA) family (TC# 2.A.1.4). In Heimdall, a homologous TC porter system (TC# 2.A.1.4.10) probably takes up both 2-phosphonoacetate and 2-phosphonopropionate [76], and a homolog of TC system 2.A.1.4.11 in MK-D1 takes up glycerol 3-phosphate [77]. In the Loki proteome, a homolog of a putative thiazole transporter (TC# 2.A.1.6.12) was found. Heimdall encodes a homolog of the 2-component, NarK1/NarK2 transport system of the Nitrate/Nitrite Porter (NNP) family (TC# 2.A.1.8). NarK1, the nitrate:proton symporter, and NarK2, the Nitrate:Nitrite antiporter, are fused in *P*. *denitificans* but are capable of functioning independently [78]. The proximity of these two proteins in the Heimdall MAG suggests that they may be capable of functioning together, thereby coupling uptake of nitrate to that of nitrite.

Thor encodes a homolog of a probable D-glucarate and/or D-galactarate:H^+^ symporter (TC# 2.A.1.14.14) [79] and, along with MK-D1, a cis, cis-muconate porter (TC# 2.A.1.15.4) [80]. Odin encodes a homolog of the proteobacterial Intraphagosomal Amino Acid Transport family (TC# 2.A.1.53). Heimdall contains an uncharacterized porter from the Microcin C51 Immunity Protein family (TC# 2.A.1.61) while Heimdall, Loki and MK-D1 encode a homolog of the putative 4-hydroxybenzoate uptake transporter (TC# 2.A.1.66.2) that may also transport S-adenosyl methionine. Loki and Heimdall each contain a homolog of the Glucose Transporter family (TC# 2.A.1.68), while Loki encodes a homolog of a niacin uptake porter (TC# 2.A.1.82.4) [81]. In addition, 11 uncharacterized homologs from 11 different, poorly characterized, TC families were present in the Asgard proteomes.

The Glycoside-Pentoside-Hexuronide (GPH):Cation Symporter family (TC# 2.A.2) is a large and ubiquitous family within the MFS. It is represented in the proteomes of Thor, Loki and MK-D1. GPH:cation symporters catalyze the uptake of a range of sugars, mostly glycosides and oligosaccharides, in symport with one or more monovalent cations (H^+^ or Na^+^) [82]. We identified homologs in Loki and MK-D1 probably capable of transporting disaccharides such as cellobiose and melibiose as well as pentosides and UDP-sugars. A single homolog of GPH was the only transport system (TC# 2.A.2.3.13) that in Thor was a likely sugar uptake system, and the majority of Loki’s sugar uptake systems were GPH homologs. Overall, small sugars, including glycosides and oligosaccharides, may be taken up primarily by members of the GPH family, especially in Loki, Thor and MK-D1.

The transport of amino acids and polyamines is mediated by porters of the Amino Acid-Polyamine-Organocation (APC) Superfamily. The APC superfamily is represented in all domains of life, and its members transport a variety of substrates by solute:cation symport or solute:solute antiport. The APC superfamily contains 15 families within 2.A.3 and 17 families outside of 2.A.3. Except for Loki, we identified APC homologs in all Asgard transportomes. MK-D1 is the only genome where we were able to identify a homolog of a eukaryotic-like APC member, the MOT2 protein (TC# 2.A.53.5.3), required for vacuolar molybdate export during senescence in *Arabidopsis* [83]. Odin is the only Asgard phylum that has a system likely to import the polyamine, putrescine (TC# 2.A.3.1.13); all other Asgard proteomes have a homolog of an MFS DHA1 antiporter (TC# 2.A.1.2.8) that exports the endogenous polyamine, spermidine [84]. In bacteria, polyamines are crucial for growth and are involved in functions such as the synthesis of siderophores, acid resistance, nucleic acid compaction and free radical scavenging [85]. Except for the aforementioned homolog of the eukaryotic molybdate transporter (TC# 2.A.53.5.3) in MK-D1, homologs of the APC superfamily present in the Asgard transportomes probably catalyze the uptake of amino acids and their derivatives [86]. Heimdall encodes a sodium-dependent tyrosine transporter homolog (TC# 2.A.22.5.2), Thor a probable amino acid uptake porter (TC# 2.A.120.1.11), and MK-D1 an adenine permease (TC# 2.A.40.7.4).

The Cation Diffusion Facilitator (CDF) Superfamily is represented by 2 different constituent families across the Asgard superphylum. The CDF Superfamily contains 8 families within 2.A.4 and 3 families outside of 2.A.4. The homologs of the CDF Superfamily, present in all five proteomes, likely maintain intracellular homeostasis of zinc, iron and manganese via the export of such ions when present at toxic levels [87, 88]. Furthermore, with the exception of Heimdall, all CDF homologs identified in Asgard showed none of the eukaryotic-specific features, such as histidine rich cytoplasmic loops [89]. Heimdall, has a homolog of the human CDF ZnT-9 transporter (TC# 2.A.4.6.1). Mutations in this transporter have been shown to cause autosomal recessive cerebro-renal syndrome and affect intracellular zinc homeostasis [90]. In addition, for all of the Asgard proteomes except for that of Odin, we identified putative Ca^2+^:Na^+^ antiport homologs of the Ca^2+^:Cation Antiporter (CaCA) family (TC# 2.A.19) [91]. Odin, Heimdall and Thor have homologs of the Ca^2+^:H^+^ Antiporter-2 (CaCA2) family (TC# 2.A.106), a family constituent of the LysE Superfamily. The CaCA2 family is ubiquitous and catalyzes the transport of calcium ions [92]. Calcium ions that may enter the cell via α-type channels may be extruded from Asgard cells by Ca^2+^:cation antiporters of either the CaCA or the CaCA2 type or both. The nine Asgard Ca^2+^ transport systems identified in this study suggests that Ca^2+^ signalling is important in these archaea as is the case for eukaryotes.

Only in Loki and MK-D1 we identified homologs most similar to prokaryotic members of the Zinc-Iron Permease (ZIP) family (TC# 2.A.5) [93]. While zinc may be taken up by these systems in Loki, it may be removed from the cell by metal-proton antiporters of the CDF family.

The Resistance-Nodulation-Cell Division (RND) superfamily (TC# 2.A.6) is represented in all Asgard proteomes due to the presence of bacterial HMG-CoA reductases (TC# 2.A.6.6.11). These systems are homologous to the C-terminal, hydrophilic domain of the essential membrane protein of the Eukaryotic Sterol Transporter (EST) family (TC# 2.A.6.6). Recognizing that these homologs lack TMSs, their functions in Asgard may not involve transport, but rather be cytosolic reductases (as annotated in NCBI). Additionally, Heimdall encodes multidrug resistance proteins of the RND superfamily within the bacterial Hydrophobe/Amphiphile Efflux-2 (HAE2) family (TC# 2.A.6.5), members of which are commonly found in Gram positive bacteria [94]. These findings reveal many of Asgard’s similarities to bacteria.

The Drug/Metabolite Transporter (DMT) Superfamily (TC# 2.A.7) is the second largest superfamily of secondary carriers represented in these Asgard proteomes. Throughout nature, this superfamily claims a range of transport proteins that can function as nutrient uptake porters, drug/metabolite efflux pumps, and solute:solute exchangers [95, 96]. Numerous homologs of the 10 TMS Drug/Metabolite Exporter (DME) family (TC# 2.A.7.3) are present in all five Asgard proteomes. The TC homologs are mostly uncharacterized or putative DME proteins. A single member of the 4 TMS Small Drug Resistance family (TC# 2.A.7.1), which is an exclusively prokaryotic family, is present in Odin. A choline uptake transporter (TC# 2.A.7.18.1) and a putative choline transporter (TC# 2.A.7.18.4) are present in Thor and Heimdall, respectively. Furthermore, four uncharacterized DMT families were represented in Odin, Thor and Loki.

Two different families of the Cation:Proton Antiporter (CPA) Superfamily, CPA1 (TC# 2.A.36) and CPA2 (TC# 2.A.37), are represented in Thor and Heimdall. These homologs resemble Na^+^:H^+^ antiporters from prokaryotes more than those from eukaryotes.

Three different family constituents of the Ion Transporter (IT) Superfamily are represented in all four Asgard phyla. This superfamily includes primary active transporters and secondary carriers that exclusively transport organic and inorganic ionic substrates [97]. Only secondary carriers of the IT superfamily were found in the Asgard phyla. All five of the Asgard proteomes possess 1–5 putative ion transporters similar to the bacterial system (TC# 2.A.45.2.2) found in magnetosome membranes of *Magnetospirillum*. These proteins are within the Arsenite-Antimonite Efflux (ArsB) family, evidencing their paralogous nature. Thor encodes homologs of a glycolate permease (TC# 2.A.14.1.2), and a putative Na^+^-coupled dicarboxylate transporter homolog (TC# 2.A.47.1.1; this last functional assignment was inferred from homology to 2.A.47.1.13).

Two of the six families in the Bile/Arsenite/Riboflavin Transporter (BART) Superfamily are represented in all 5 proteomes of Asgard. Homologs from the Arsenical Resistance-3 (ACR3) family (TC# 2.A.59) were identified in these Asgard phyla, except for Thor. These ACR3 homologs may confer resistance to the toxic metalloid arsenite [98]. Uncharacterized homologs (TC# 2.A.69.4.3) within the Auxin Efflux Carrier (AEC) family is encoded in the Thor and Heimdall MAGs. Homologs of families ArsB (a member of the IT superfamily) and ACR-3, encoded within these Asgard phyla, are likely the major detoxification systems for arsenic via the extrusion of arsenite [98, 99].

Three different constituent families of the Multidrug/Oligosaccharidyl-lipid/Polysaccharide (MOP) Flippase Superfamily (TC# 2.A.66) are represented in Odin, Loki and MK-D1. All three transportomes contain uncharacterized homologs of the Polysaccharide Transport (PST) family (TC# 2.A.66.2), while Odin and MK-D1 have homologs of the uncharacterized MOP-12 (U-MOP12) family (TC# 2.A.66.12). Odin has the only Asgard MAG where we detected homologs of the Putative Exopolysaccharide Exporter (EPS-E) family (TC# 2.A.66.6). EPS-E homologs in Odin may play roles in biofilm formation via the export of exopolysaccharides [100].

Six of the eleven families in the L-Lysine Exporter (LysE) Superfamily [101] are represented within the Asgard phyla. Loki, Thor and MK-D1 have putative amino acid efflux transporters of the Resistance to Homoserine/Threonine (RhtB) family (TC# 2.A.76). RhtB homologs may function in the regulation of intracellular levels of neutral amino acids by catalyzing efflux of these compounds [102]. Loki’s and MK-D1’s proteomes are the only ones to contain a putative manganese exporter of the Mn^2+^ Exporter (MntP) family (TC# 2.A.107). Ferrous iron uptake is likely mediated by the porters of the Iron/Lead Transporter (ILT) family (TC# 2.A.108) that were identified in the proteomes of Heimdall, Thor and Loki. However, in all four phyla, ferrous iron uptake is mediated by FeoB transporters of TC family 9.A.8. Homologs of the Nickel/Cobalt Transport (NicO) family (TC# 2.A.113) were identified in the proteomes of Heimdall, Loki and MK-D1, respectively.

Five of the twelve families from the Transporter-Opsin-G Protein-coupled Receptor (TOG) Superfamily are represented within these Asgard proteomes. Loki encodes a high affinity Ni^2+^-specific transporter [103] of the Ni^2+^-Co^2+^ Transporter (NiCoT) family (TC# 2.A.52). All Asgard proteomes include 1–4 paralogs of a putative permease of the 4-Toluene Sulfonate Uptake Permease (TSUP) family (TC# 2.A.102). Although the TSUP family has not been rigorously characterized, bioinformatic approaches have shown that the TSUP family is ubiquitous in nature and often mediates the uptake of sulfur-containing compounds [104]. Heimdall and MK-D1 contain a putative transporter homolog of the Lipid-linked Sugar Translocase (LST) family (TC# 2.A.129). In MK-D1 we found one homolog each of the Organo-Arsenical Exporter (ArsP) family (TC# 2.A.119) and the HelioRhodopsin (HelioR) family (TC# 3.E.3).

**Fig 2:**
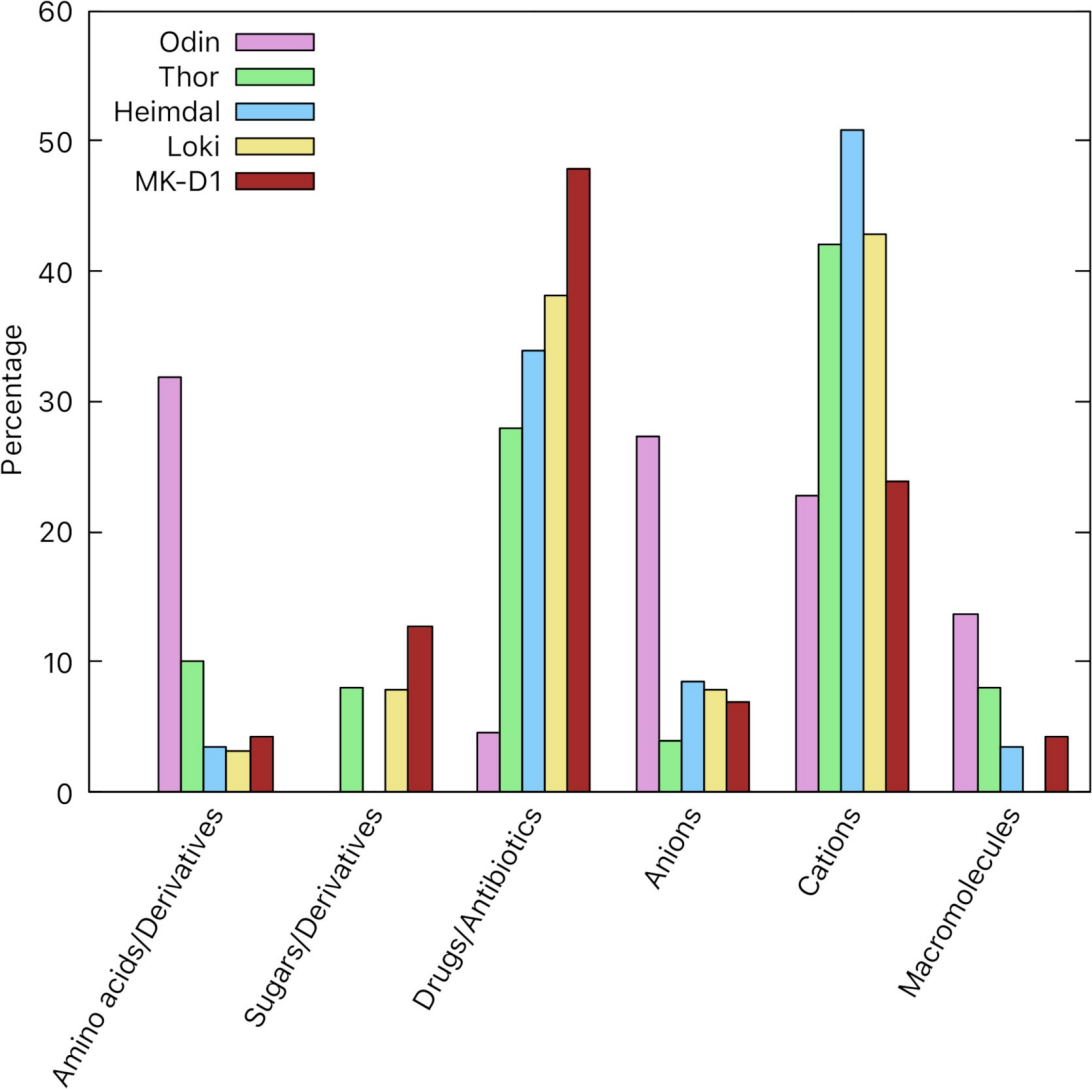
Inferred substrates transported by secondary carriers (subclass 2.A) included within the five Asgard transportomes. The percentage of each substrate type is calculated relative to the total number of substrates transported by secondary carriers per Asgard transportome.

Asgard porters from TC subclass 2.A transport a diverse range of substrates, as summarized in Fig 2. The most common substrates transported by this subclass are cations, drugs, and anions. Odin is the only MAG where we could identify more anion porters than cation porters. Anions transported by Asgard include inorganic anions such as phosphate, arsenite, and chloride, and organic anions such as pyruvate and lactate. The cations transported by the porter subclass are mostly inorganic cations such as sodium, hydronium ions, and various heavy metals. Heimdall and Thor have orthologs of an organic cation porter, which mediates the transport of the substrate, choline. Polyamines may be exclusively transported in Asgard by secondary carriers. Contrasting with the other proteomes, the largest substrate category in Odin for secondary carriers is amino acids and their derivatives.

### Pyrophosphate hydrolysis-driven primary active transporters (TC subclass 3.A)

#### The ABC superfamily (TC superfamily 3.A.1)

The best represented primary active transport superfamily in Asgard is the ATP Binding Cassette (ABC) Superfamily (TC# 3.A.1). Members of the ABC superfamily are found in all domains of life and include proteins responsible for ATP hydrolysis-driven translocation of molecules across the cell membrane, for either uptake or export [105]. Of the Asgard transportomes, Heimdall (20%) and Thor (18%) contain percentagewise the most ABC transport systems, while Loki (9.7%) and MK-D1 (12.2%) contain the least. In Odin, 15.3% of the transport systems belong to the ABC superfamily with more ABC uptake systems than efflux systems.

**Fig 3:**
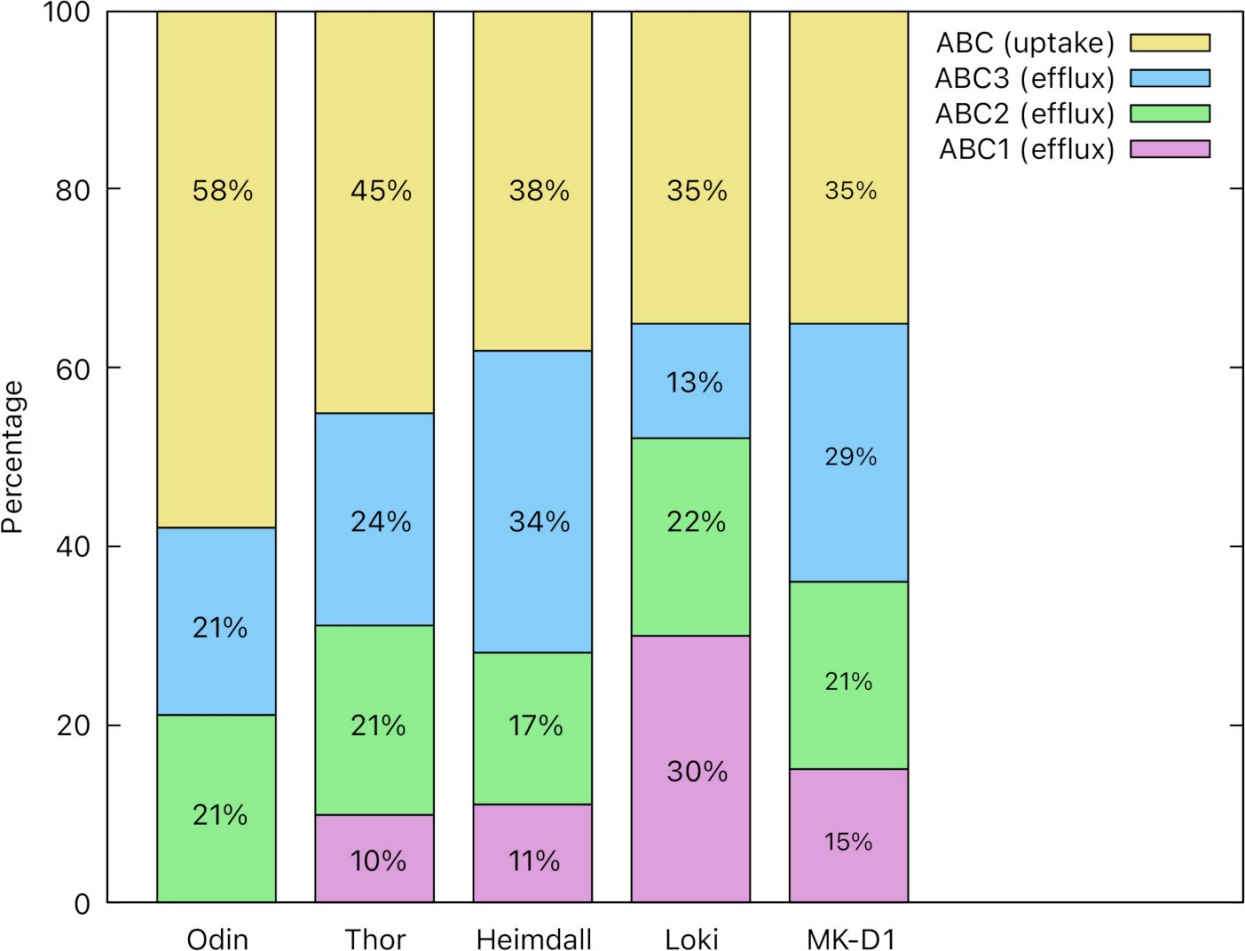
ABC types encoded within the five Asgard proteomes. The percentages of efflux and uptake ABC transporters are shown for all Asgard proteomes. Note that a) efflux systems predominate in all Asgard proteomes, b) ABC1 efflux systems were not detected in Odin, and c) Loki has the only MAG in which we identified more ABC1 systems than ABC2 or ABC3 systems.

According to TCDB, ABC exporters are divided into three types (ABC1, ABC2, ABC3) based on the distinct evolutionary paths of their integral membrane proteins [106]. Type 1 ABC transporters (ABC1) were not found in Odin and were the least represented ABC type in all Asgard proteomes, except that of Loki (Fig 3). ABC1 exporters in these Asgard proteomes are all multidrug exporter homologs, except for one homolog in Heimdall which is a lipid precursor exporter (TC# 3.A.1.106.14) apparently essential for acid, salt, and thermal tolerance [107]. ABC3 exporters are the second largest group of ABC efflux porters encoded within these transportomes, except for Loki where it is the least populous type of ABC system. Aside from peptide exporters of the peptide-7 Exporter (Pep7E) family (TC# 3.A.1.134) represented in Heimdall and MK-D1, the rest of the ABC3 transporters present in Asgard hit uncharacterized systems in TCDB. In *Staphylococcus aureus*, the Pep7E homolog forms a five-component system with GraXSR sensing cationic antimicrobial peptides (CAMP), signaling to downstream enzymes to confer resistance to such peptides [108]. However, it should be noted that the GraXSR system and systems that confer resistance to antimicrobial peptides such as MprF (TC# 4.H.1.1.1) were not found in the Asgard proteomes, and therefore CAMP resistance may not be a characteristic of these organisms. Homologs of the Macrolide Exporter family (TC# 3.A.1.122; ABC3) are present in all Asgard proteomes, except that of Loki. However, none of the members of the Macrolide Exporter family in TCDB that most closely resemble the Asgard proteins have been characterized functionally. Heimdall contained ABC3 transport systems showing extensive sequence divergence from other ABC3 proteins in Asgard, and they were therefore added to TCDB as a new uncharacterized TC family (TC# 3.A.1.157). We were able to identify homologs of the Eukaryotic ABC3 (E-ABC3) family (TC# 3.A.1.207) in Heimdall and MK-D1.

Of the efflux systems, ABC3 transporters are the best represented ABC type in these Asgard proteomes (except in Loki), followed by ABC2. Uncharacterized and drug/antimicrobial peptide exporters make up the majority of the ABC2 efflux systems in the Asgard phyla. Proteins of the Drug Exporter-1 family (TC# 3.A.1.105) are present in all Asgard proteomes and are the best represented group of ABC2 efflux homologs in Thor and Odin. Examples of substrates of these drug exporters are chromomycin and pyoluteorin in Odin and a putative lantibiotic exporter in Heimdall. Other ABC2 efflux systems are homologs of putative sodium exporters in Heimdall and Thor and a putative heme exporter in Loki. The most commonly recognized functions of the ABC efflux systems in Asgard are drug and antimicrobial peptide export, suggesting that multidrug resistance is a major function of ABC transporters in the Asgard superphylum.

ABC uptake transporters in Asgard make up the majority of the ABC transport systems [106, 109]. Homologs of the Peptide/Opine/Nickel Uptake Transporter (PepT) family (TC# 3.A.1.5) are present in all four metagenomes. These PepT homologs in Asgard are peptide uptake systems with one exception, a probable cellobiose uptake porter (TC# 3.A.1.5.14) in Thor. Homologs from the Sulfate/Tungstate Uptake Transport (SulT) family (TC# 3.A.1.6) were found in the MAGs of Odin, Heimdall and Loki. SulT homologs present in Asgard presumably mediate the uptake of tungstate and vanadate, cofactors for enzymes generally involved in the transformation of carbon-, nitrogen-, and sulfur-containing compounds [110, 111].

**Fig 4:**
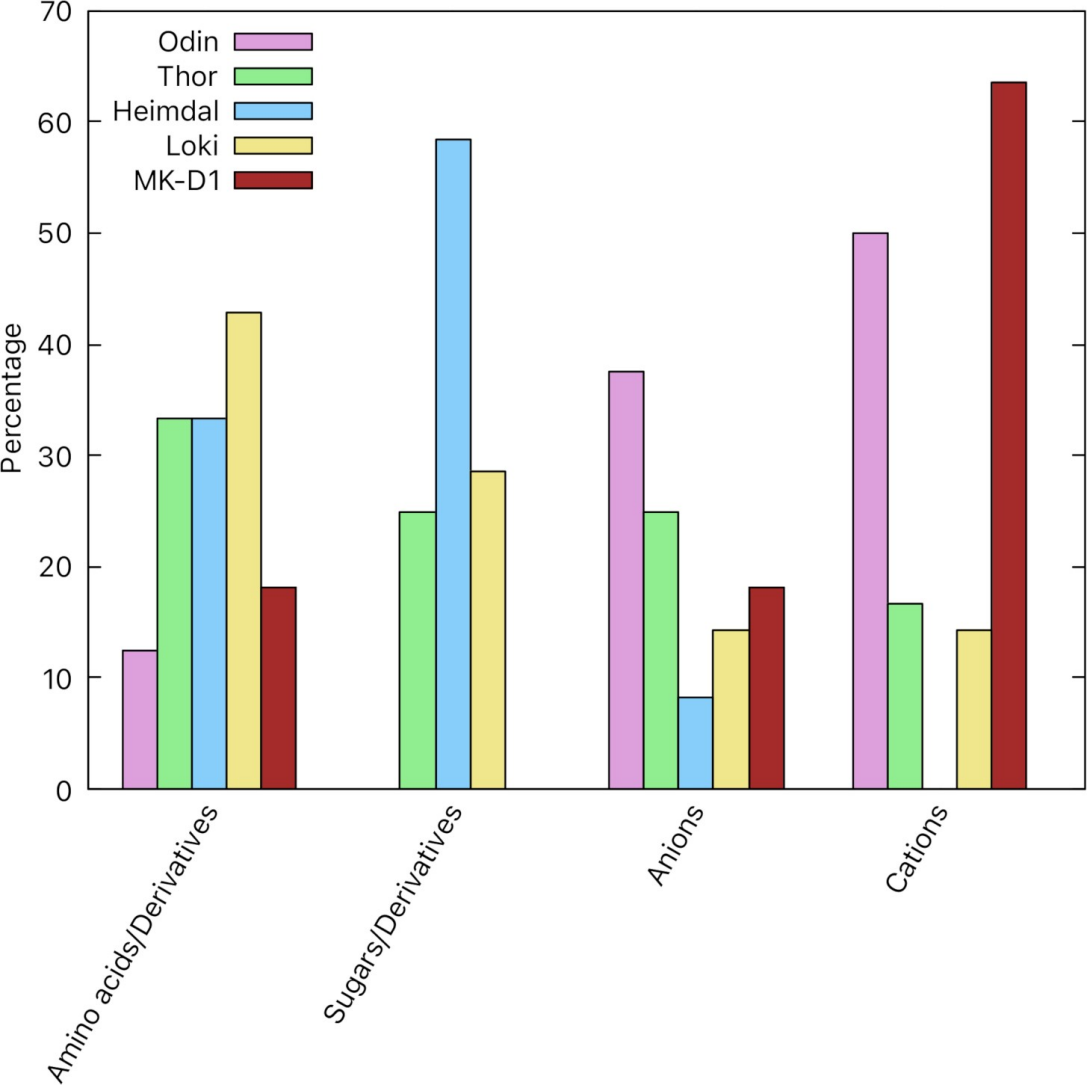
Substrates imported by ABC systems encoded within the five Asgard transportomes. The percentage of each substrate type was calculated relative to the total number of substrates transported by ABC uptake systems per Asgard transportome.

Fig 4 shows that 50% of the substrates imported by ABC uptake systems in Odin and 64% in MK-D1 are cations, including metal ions such as Cobalt, Zinc, Nickel, and Manganese. In Heimdall, 58% of the inferred substrates imported by ABC uptake systems are sugars and sugar derivatives, but in Odin and MK-D1, we were unable to identify ABC systems that import sugars and sugar derivatives.

#### Additional major families of pyrophosphate hydrolysis-driven active transporters

Table 2 shows that pyrophosphate hydrolysis-driven transporters (TC subclass 3.A), including ABC superfamily members, are present in all proteomes, but Odin has at least, ~50% fewer than the others. Given that we identified almost twice as many secondary carriers as primary pyrophosphate bond hydrolysis-driven transporters in Loki and MK-D1, these organisms may rely more on the uptake and export of nutrients and toxins by secondary carriers than by phosphoryl bond hydrolysis-driven primary active transporters, suggesting greater reliance on *pmf*-generating electron flow than on substrate-level phosphorylation.

The H^+^- or Na^+^- translocating F-type, V-type, and A-type ATPase (F-ATPase) Superfamily (TC# 3.A.2) was found in all Asgard transportomes. These ATPases are all proton pump complexes that can synthesize ATP when the rotor complex is driven by a proton motive force (*pmf*). Conversely, these pumps can hydrolyze ATP to export protons across the membrane to establish a *pmf* [112]. Odin, Thor and Heimdall contain homologous sets of the integral membrane protein system of the archaeal (A-type) ATP synthase (TC# 3.A.2.3.2). These A-type complexes can use Na^+^ or H^+^ gradients to synthesize ATP [113]. The presence of A-type ATPases and the establishment of two ion gradients (i.e., proton and sodium) is a characteristic that has only been observed in methanogens [113, 114]. However, the Loki MAG is the only one where we identified both an archaeal A-type ATPase (TC# 3.A.2.3.1) and a eukaryotic-like V-type ATPase (TC# 3.A.2.2.8). The eukaryotic-like V-type ATP synthase present in Loki is uncharacterized, but due to homology, it probably transports protons and possibly other ions such as sodium and lithium as well [115]. This V-type ATPase is unique to eukaryotes and provides support for Loki’s proposed ancestral relationship to eukaryotes. MK-D1 contains a bacterial-like H^+^-translocating V-type ATPase (TC# 3.A.2.2.1), for which cryo-EM structures in *Thermus thermophilus* show substantial flexibility between V_1_ and V_0_ that results from mechanical competition between central shaft rotation and resistance from the peripheral stalks [116].

The P-type ATPase (P-ATPase; TC# 3.A.3) Superfamily is represented by various homologs throughout the Asgard proteomes, and its members mediate uptake and efflux of cations driven by ATP hydrolysis [117]. All five Asgard proteomes include orthologs of a putative Ca^2+^ ATPase (TC# 3.A.3.2.21), again supporting the notion that Ca^2+^ signaling is important in these organisms as is true for eukaryotes. A variety of members of the Copper Cation P-ATPase family (TC# 3.A.3.5) are present in these Asgard proteomes. These homologs in Asgard archaea may confer copper resistance by maintaining low levels of intracellular copper [118].

All five Asgard transportomes encode the essential components of the General Secretory Pathway (Sec) family (TC# 3.A.5). Sec protein complexes are found universally in all 3 domains of life and are responsible for protein secretion and integration into membranes [119]. The archaeal Sec complexes consist of a large transmembrane SecYEG complex and the ribosome-associating signal recognition particle (SRP) complex. The SRP complex binds to nascent polypeptides that are destined for secretion or membrane insertion as they emerge from translating ribosomes [120]. The newly formed SRP/ribosome nascent chain complexes are then targeted to the membrane embedded receptor, FtsY. The nascent polypeptide is transferred to the SecYEG protein translocon complex that either secretes the polypeptide or inserts it into the membrane [121].

Homologs of the H^+^ or Na^+^-translocating Pyrophosphatase (M^+^-PPase) Family (TC# 3.A.10) are present in all Asgard proteomes. Heimdall, Thor, and Odin contain homologs of a H^+^-translocating PPase (TC# 3.A.10.2.2) while Na^+^-translocating PPases were identified in Loki (TC# 3.A.10.1.3) and MK-D1 (TC# 3.A.10.1.5). Therefore, these homologs may contribute to *pmf* and *smf* generation via proton, or in the case of Loki and MK-D1, sodium extrusion, coupled to pyrophosphate hydrolysis [122, 123].

Orthologous proteins of the Guided Entry of Tail Anchored Protein (GET) family (TC# 3.A.19) are present in all Asgard metagenomes. This suggests the presence of a novel membrane protein-targeting pathway in archaea [124].

### Oxidoreduction driven transporters (TC subclass 3.D)

In accordance with previous research [36], we identified two transport systems of the H^+^- or Na^+^-translocating NADH Dehydrogenase (NDH) Family (TC# 3.D.1) in Odin. Although the following transport systems are members of the NDH family, they appear not to be NADH dehydrogenases. The first system is a [Ni^2+^-4Fe-4S] quinone-independent ferredoxin:H^+^ oxidoreductase (TC# 3.D.1.4.2) that translocates protons for *pmf* generation [125]. Odin has the only MAG in which we could identify a transport complex capable of reducing ferredoxin, an essential step in methanogenesis. The second system in Odin is the formate-dependent [NiFe] hydrogenase (TC# 3.D.1.9.2) that may couple H_2_ production with oxidation of formate to carbon dioxide [126]. Homologs of the H_2_:Heterodisulfide Oxidoreductase (HHO) Family (TC# 3.D.7) were identified in Heimdall (TC# 3.D.7.1.1). HHO systems are used in cytochrome containing methanogenic archaea to generate a *pmf* using redox-driven proton extrusion concomitant with the reduction of a heterodisulfide [127–129]. Homologs of another HHO system (TC# 3.D.7.1.5) that seems to lack the integral membrane-bound protein are present in all proteomes, except for Odin. Interestingly, tungsten, which is imported via an ABC uptake system (TC# 3.A.1.6.2), is an important cofactor for the reduction of CO_2_ to CH_4_ [110].

Homologs of the Na^+^ or H^+^ Pumping Formyl Methanofuran Dehydrogenase (FMF-DH) family (TC# 3.D.8) were identified in Odin, Thor and Loki. FMF-DH couples Na^+^ transport with the initiation of methanogenesis using the archaeal cofactor, methanofuran [130]. Odin, Thor and Heimdall contain orthologous sets of proteins from the H^+^-translocating F_420_H_2_ Dehydrogenase (F_420_H_2_DH) family (TC# 3.D.9). The F_420_H_2_DH proteins form a redox-driven proton pump complex that, in methanogenic archaea, couples the reduction of methylated compounds to proton extrusion [131]. Seitz et al. [23] suggested that the F_420_H_2_DH in Thor likely performs non-methanogenic functions due to the lack of other genes required for methanogenesis. However, it should be pointed out that their suggestion has not been verified because no fully sequenced genomic data were available at the time of their study.

Orthologous sets of the Prokaryotic Succinate Dehydrogenase (SDH) family (TC# 3.D.10) were found in Thor, Heimdall and Loki. The E succinate:quinone reductase (SQR) systems (TC# 3.D.10.1.4) of the SDH family contain a [4Fe-4S] cluster in reduced (2+) and oxidized (3+) states in *Sulfolobus solfataricus* [132]. The SQR system generates and utilizes a transmembrane electrochemical proton potential by means of transmembrane electron transfer coupled to reactions on opposite sides of the membrane, which was first demonstrated for the *pmf*-dependent catalysis of succinate oxidation by quinone in the case of the diheme-containing SQR enzyme from the Gram-positive bacterium *Bacillus licheniformis* [133].

Examination of the Asgard MAGs revealed the presence of diverse means of generating a *pmf* for subsequent ATP generation or electrochemical driven transport. Due to the presence of enzymes that could confer upon Odin the ability to ferment organic substrates to formate [36], formate-dependent NDH-like systems may allow Odin to use the products of fermentation to enhance *pmf* generation. We identified transport systems in Odin that are essential for methanogenesis in methanogens without cytochromes [134]. Odin possesses a hydrogenase 3 (TC# 3.D.1.9.2) that in *E*. *coli* forms an H_2_-dependent redox-driven proton pump and may convert methylated compounds to CO_2_ and H_2_ for subsequent reduction to produce methane [131, 135]. Additionally, CO_2_ produced by formate oxidation may be used for methane production. However, the membrane associated methyl-H4MPT–coenzyme M methyltransferase (TC# 3.C.1.1.1), which is an essential membrane component for the reduction of carbon dioxide to methane [134, 136], was not found in any of the five Asgard proteomes. Odin has the only Asgard MAG to lack homologs of the HHO family that are present in all methanogens [114, 131, 134]. The evidence suggests that Odin contains the initial membrane components required for the utilization of methylated compounds and formate for methanogenesis but may lack the components necessary for the reduction of carbon dioxide to methane. Therefore, the present oxidoreduction-driven transporters suggest that Odin’s ability to generate a *pmf* is multifaceted and relies on various electron acceptors such as formate, hydroxy-phenazine, and ferredoxin. However, the potential for methanogenesis cannot be ruled out because the methyltransferase function may be carried out by an unidentified transport system. In addition, while Odin contains some of the essential methanogenic transport complexes, there is no evidence for the presence of the various cytosolic enzymes required for methanogenesis [36]. However, further investigation of more DNA sequence data for Odin will be required in order to gain support for this suggestion.

Loki and Thor both contain oxidoreduction-driven transport systems like those of methanogens without cytochromes [134]. Consistent with previous findings [36], non-membrane associated HHO systems (TC# 3.D.7) were found in Thor, Loki and MK-D1, which is another characteristic of methanogens that lack cytochromes. Odin, Thor and Loki contain FMF-DH proton pumps that are required for the utilization of methylated compounds for methanogenesis [130]. However, MtrA-H and ferredoxin hydrogenase, which are essential to methanogenesis, were not found in any of the Asgard proteomes. Like Odin, the oxidoreduction driven transport systems in Loki and Thor probably do not play a role in methanogenesis but contribute to the production of a *pmf* using electron acceptors such as fumarate and organic heterodisulfides.

Heimdall contains some membrane components involved in methanogenesis, similar to those of the order Methanosarcinales. This type of methanogen forms an electrochemical gradient using cytochromes, as opposed to other methanogens that do not contain cytochromes, and the process involves redox-driven ion translocation across the membrane that is catalyzed by an anaerobic respiratory chain [131]. Heimdall has the only Asgard proteome that contains the key membrane components of methanogens with cytochromes, such as a transport system of the Proton-translocating Cytochrome Oxidase (COX) Superfamily (TC# 3.D.4) and the membrane‐bound heterodisulfide reductase of the HHO family (TC# 3.D.7.1.1). The membrane constituents of the cytochrome-containing methanogens of the Methanosarcinales are present except for the MtrA-H system and the ferredoxin oxidoreductase. In addition, the homologous COX transport system in Heimdall (TC# 3.D.4.4.4) is probably an oxygen-dependent proton pump, and therefore may not play a role in methanogenesis [137]. This suggestion supports previous research identifying Heimdall as a possible facultative anaerobe [35, 36, 138]. Our findings indicate that it is unlikely that Heimdall is capable of methanogenesis, but it may use oxygen, fumarate, and organic heterodisulfides as final electron acceptors.

No oxidoreduction-driven transport system was found to be ubiquitous to all Asgard proteomes, but there are possible homologous transport systems encoded within three of the five transportomes. Therefore, it was difficult to determine the origin of these genes and the overarching means of *pmf*-driven ATP synthesis within the Asgard superphylum. All Asgard proteomes studied here seem to depend primarily on generating a *pmf* using protons rather than sodium ions and depend on a variety of transport systems unique to methanogens. However as noted above, we were unable to confidently establish the capability for methanogenesis due to incomplete metabolic and transport pathways. The *pmf* generated is likely used to drive ATP synthesis and proton gradient-driven transport by secondary carriers.

### Group translocators (TC class 4)

Group translocators are more common in Loki and MK-D1 than in the other Asgard transportomes, mostly due to the presence of homologs of the Fatty Acid Group Translocation (FAT) Family (TC# 4.C.1). The numbers of FAT homologs are variable across the four Asgard proteomes, with Odin containing just 1 homolog and Loki containing 25 homologs. Research has shown that the FAT family’s role in linking the translocation of fatty acids with acylation by CoA to form thioesters can be distinct as well as overlapping [139–141]. Asgard proteomes encode multiple homologs of putative FAT systems and show sequence similarity to some well characterized FAT systems that reportedly couple translocation to esterification. The most abundant FAT systems in all transportomes except that of Odin, is the long chain fatty acyl CoA synthase (TC# 4.C.1.1.4). These homologs may have been selected for via gene duplication events in response to natural pressures. This may indicate the vital physiological role that FAT transport proteins may have in Loki. As observed in *E*. *coli*, these active CoA thioesters may be degraded or incorporated into phospholipid membranes in Thor, Heimdall, Loki and MK-D1 [140, 142]. Interestingly, ester-linked phospholipids are unique to eukaryotes and bacteria while ether-linked phospholipids are characteristic of archaea [143]. This raises the question as to whether esterified phospholipids are produced in Asgard, and if they make up parts of the phospholipid bilayer, like those of bacteria and eukaryotes, or if they are merely degraded as a source of carbon and energy.

Three different families within the Polysaccharide Synthase/Exporter (TC# 4.D) subclass are represented in these Asgard proteomes. Two of these families (TC# 4.D.1 and 4.D.3) belong to the Glycosyl Transferase/Transporter Superfamily. Homologs from the Putative Vectorial Glycosyl Polymerization (VGP) Family (TC# 4.D.1) were identified in Odin, Heimdall and MK-D1. These proteins may couple the export of glycosyl groups when added to growing polysaccharide chains [144, 145]. Furthermore, these glycosyl transferase homologs may be essential for biofilm formation and intercellular adhesion [146]. A possible cellulose synthase (TC# 4.D.3.1.2) and a putative eukaryotic-like integral membrane glycosyl transferase homolog (TC# 4.D.3.1.10) of the Glycan Glucosyl Transferase Family (TC# 4.D.3) were only found in Heimdall.

All Asgard proteomes, except that of Odin and MK-D1, contain homologs of the Glycosyl Transferase 2 family (TC# 4.D.2). Multiple proteins from Thor, Heimdall and Loki are homologous to the same putative glycosyl transferase (TC# 4.D.2.1.6), which may function as a glycosyl transferase and a membrane transporter.

A range of 1–6 homologs of the Choline/Ethanolamine Phosphotransferase 1 (CEPT1) family (TC# 4.F.1) are present in all Asgard proteomes. All of them include at least one homolog of the CDP-alcohol phosphatidyl transferase (TC# 4.F.1.3.1). This system is uncharacterized, but it is inferred by sequence similarity to play a role in phospholipid synthesis and insertion into the membrane through the displacement of CMP from a CDP-alcohol by a second alcohol [147].

### Transmembrane electron carriers (TC class 5)

Homologs from the Disulfide Bond Oxidoreductase D (DsbD) Family (TC# 5.A.1), a constituent family of the LysE superfamily, are present in all Asgard proteomes, except that of Odin. Thor, Heimdall and Loki and MK-D1 contain 1–3 homologs of CcdA within subfamily 5.A.1.2. CcdA proteins shuffle electrons into the periplasm from the cytoplasm via 2 cysteines, cycling between oxidized and reduced states [148]. Heimdall encodes homologs of the Prokaryotic Molydopterin-containing Oxidoreductase (PMO) family (TC# 5.A.3), which are usually multi-component DmsABC(E) systems (TC# 5.A.3.3.3) as well as one NarGHI system (TC# 5.A.3.1.1). These systems may contribute to the *pmf* across its cell membrane in Heimdall. This suggests that Heimdall is capable of proton translocation and reduction of dimethyl sulfoxide/trimethylamine N-oxide and nitrate via 2 different systems, DmsC [149] and NarA [127], respectively. The single one-electron transmembrane carrier homolog identified in Loki may be essential for the utilization of phenol as a carbon source [150].

Compared to the other Asgard proteomes, Heimdall has at least 3 times more transmembrane electron carrier systems (Table 2). Transmembrane electron carriers can shuttle electrons from electron donors in the cytoplasm to recipient substrates in the external milieu or vice versa, thus affecting membrane potentials. These homologs likely introduce reducing equivalents from the cytoplasm to the periplasm of Gram-negative bacteria to facilitate essential reducing pathways [151]. Reduced substrates in the external environment of Thor, Heimdall, Loki, and MK-D1 may promote correction of non-native disulfide bonds, defense against oxidative damage, and cytochrome c biogenesis [151, 152].

### Auxiliary transporters (TC subclass 8.A)

TC subclass 8.A contains proteins that facilitate transport, but do not participate directly in the transport process. They function in conjunction with one or several established TC systems. The Voltage-gated K^+^ Channel β-subunit (Kvβ) family (TC# 8.A.5) is represented in all Asgard proteomes. However, their presence does not necessarily imply that this is their function in Asgard organisms. Five other families from TC subclass 8.A were found to be present in 50% or less of the Asgard proteomes. One such family is the Stomatin/Podacin/Band7/Nephrosis.2/SPFH (Stomatin) Family (TC# 8.A.21) that is represented in Thor and Heimdall. At least three homologs of auxiliary transport proteins of the Kvβ family are present in Heimdall, Loki and MK-D1. In vitro analyses have shown that these homologs may function in deactivation of voltage-gated K^+^ channels [153]. However, as mentioned earlier, voltage-gated K^+^ channels were only identified in Heimdall and MK-D1. Therefore, these proteins may be ubiquitous oxidoreductases that function as bacterial stress response proteins. The stomatin homologs in Heimdall and Thor are membrane-bound proteases that may function to open ion channels [154].

### Poorly characterized transporters (TC subclass 9.A)

Proteins in this subclass function in transport across a membrane via an unknown mechanism. Homologs from the Ferrous Iron Uptake (FeoB) family (TC# 9.A.8) are present in all Asgard proteomes. Thor also contains two uncharacterized homologs of the Mitochondrial Cholesterol/Porphyrin Uptake Translocator Protein family (TC# 9.A.24).

FeoB homologs represent the majority of proteins in this subclass. These homologs import ferrous iron, which is predominantly present in highly acidic, reducing, and anaerobic environments and cannot be taken up complexed to siderophores. The uptake of ferrous iron is possibly mediated in a process powered or regulated by the action of GTP binding proteins [155]. The FeoB proteins present in Asgard are of the prokaryotic type and likely associate with GTPases encoded nearby in the DNA [3].

### Putative transport proteins (TC subclass 9.B)

Proteins in this subclass are either awaiting classification upon elucidation of function or will be deleted from TCDB if their suggested transport function is disproven. This subclass is the second largest group of systems in the Asgard proteomes. Homologs from the Integral Membrane CAAX Protease (CAAX protease) family (TC# 9.B.1) and the Integral Membrane CAAX Protease-2 (CAAX Protease-2) family (TC# 9.B.2), belonging to the CAAX Superfamily, are present in all Asgard transportomes. CAAX proteases have been shown to cleave and degrade transmembrane α-helices anchored in the cell membrane [156, 157]. Putative heme exporters [158, 159] of the Heme Handling Protein family (TC# 9.B.14) were identified in Asgard proteomes, except that of Thor. The Integral Membrane Glycosyltransferase 39 (GT39) family (TC# 9.B.142) is present in all Asgard proteomes. The GT39 family may be distantly related to other glycosyl transferases (TC# 4.D.1, 4.D.2). Homologs of the eukaryotic GT39 system (TC# 9.B.142.3.3) were found in all five Asgard proteomes.

The following families are represented in all Asgard proteomes, unless stated otherwise: The DedA family (TC# 9.B.27) is represented in all except Heimdall. We also identified homologs of the Acid Resistance Membrane Protein family (TC# 9.B.36), the Acyltransferase-3/Putative Acetyl-CoA Transporter family (TC# 9.B.97), the Lipoprotein Signal Peptidase/Phosphatase/Lead Resistance Fusion Protein family (TC# 9.B.105), the Putative Integral Membrane Steroid 5α-reductase (SαR) family (TC# 9.B.115), the Putative Undecaprenyl-phosphate N-Acetylglucosaminyl Transferase family (TC# 9.B.146), the M50 peptidase family (TC# 9.B.149), the Prenyl Transferase family (TC# 9.B.241), and three uncharacterized families (TC# 9.B.288; 9.B.289; 9.B.303).

### Major superfamilies

**Fig 5:**
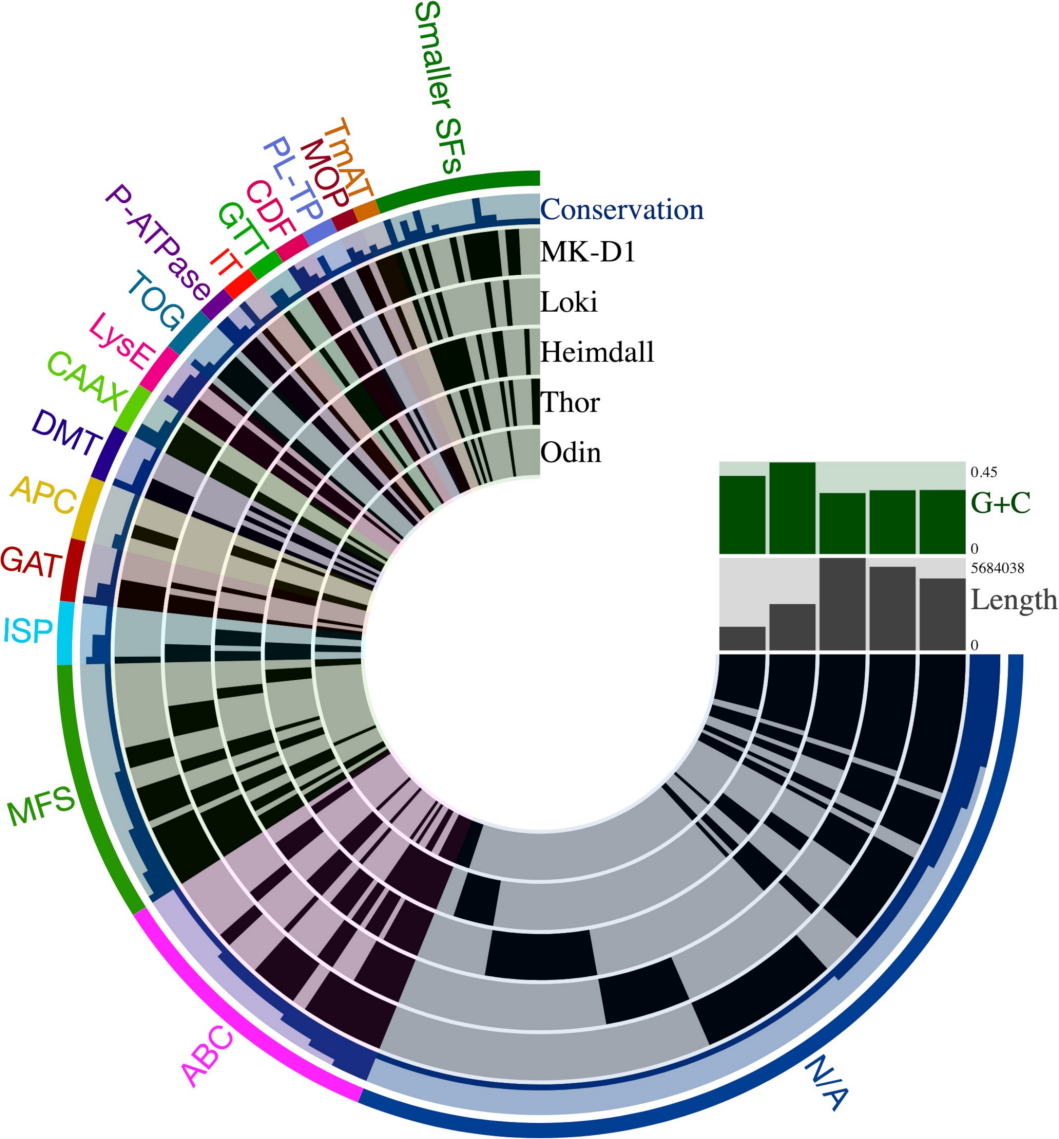
Conservation of superfamilies within five Asgard proteomes. TC Subfamilies were selected as the basic unit for counts across all proteomes. Black bars in the first five layers from the center indicate the presence of individual transport subfamilies. The patterns formed by these bars convey the presence or absence of individual subfamilies across proteomes. Blue bars in the sixth layer (Conservation) show the number of proteomes that contain at least one member of a particular subfamily. The outermost layer labels superfamilies as annotated in TCDB. Superfamilies are organized clockwise by decreasing size based on the number of different subfamilies the proteomes contain. Label “Smaller SFs” refers to superfamilies with fewer subfamilies. Label “N/A” denotes subfamilies not yet assigned to any superfamily. This figure was generated using Anvi’o [160, 161].

In TCDB, Superfamilies are composed of sequence divergent families that are derived from a common ancestral system and often share similar functions, determined by several conserved characteristics such as topology, protein domains and motifs (see Methods). The distribution of the major superfamilies within the Asgard metagenomes are shown in the outermost layer of Fig 5. It is evident that the majority of Asgard transport systems belong to families that have not been assigned (N/A) to any superfamily. For example, 52% of putative transporters conserved across all four Asgard metagenomes belong to this N/A group. Also note that some highly conserved systems in the N/A group include numerous paralogs of the FAT (TC# 4.C.1) and SαR (TC# 9.B.115) families, which suggests that they are important to the survival of these organisms. A substantial fraction of subfamilies (40–73%) present in only one of the five proteomes within the N/A group belong to TC subclass 9.B of putative transporters of uncharacterized function, thus emphasizing both their diversity and the current gap in knowledge about Asgard transport systems.

Fig 5 shows that the ABC and the MFS are the largest superfamilies represented within the five Asgard proteomes examined here. Loki and MK-D1 are the only proteomes where we identified more MFS transport proteins than ABC transport proteins. Within the ABC superfamily, the transport homologs present in largest numbers across all proteomes are: the peptide/oligosaccharide uptake systems (TC# 3.A.1.5), membrane-associated ATPases of the ABC superfamily that are essential for mRNA to protein translation and are ubiquitous among eukaryotes and archaea (TC# 3.A.1.31), peptide antibiotic efflux systems (TC# 3.A.1.105), and two different types of uncharacterized exporters. Thor and MK-D1 encode homologs of a bacterial ABC uptake system (TC# 3.A.1.16.2) specific for nitrite and cyanate of the Nitrate/Nitrite/Cyanate Uptake Transporter (NitT) family (TC# 3.A.1.16). While Heimdall also has the capability to import nitrite/nitrate, through an MFS porter (TC# 2.A.1.8.11), we suggest that Thor and MK-D1 have the unique capability to assimilate cyanate at low nitrite concentrations via the NitT ABC-2 system (TC# 3.A.1.16.2), as observed in *Synechococcus elongatus* [162].

Drug:H^+^ antiporters are the most conserved homologs within the MFS, indicated by their conservation within the Asgard proteomes and the average number of homologs identified per proteome, that is, 9.4 for DHA1 (TC# 2.A.1.2) and 4.8 for DHA3 (TC# 2.A.1.21). The 10 TMS Drug/Metabolite Exporter (DME) family (TC# 2.A.7.3) is the largest family within the DMT superfamily, accounting for about 76% of the transport systems within this superfamily. The largest number of DME systems present within the Asgard archaea are most similar to an archaeal uncharacterized transport system (TC# 2.A.7.3.5). Homologs of the LysE superfamily are present in all Asgard proteomes, but no one family was found to be conserved in all.

### Substrates

To gain a better understanding of Asgard’s physiology and its interactions with its surrounding environments, substrates were inferred based on TCDB annotations for identified systems (see Methods). Table 3 shows that approximately half of the transport systems in the five Asgard proteomes are of unknown function. Individual substrate assignments per TC system are available in S1 Table. The largest group of predicted substrates transported are cations, mostly hydronium ions (protons) and inorganic cations. These cations, such as copper and calcium, may function as enzyme cofactors and signaling molecules. Furthermore, protons likely play a large role in generating electrochemical potentials and driving ATP synthesis. With respect to drug and antimicrobial agent exporters, Odin contains the fewest while MK-D1 contains the largest numbers of these systems (Table 3). For lipid precursor transporters, Loki contains more than any one of the other four Asgard proteomes. The transported lipid precursors include diterpenes and fatty acids. For systems transporting amino acids and their derivatives, Loki and MK-D1 encode the fewest (7 to 10) mainly moving peptides and alpha amino acids across the membrane. Notably, no recognized sugar transporters were found in Odin.

**Table 3:** Inferred substrates transported by Asgard archaea.

Substrate	ChEBI	Odin	Thor	Heimdall	Loki	MK-D1
**Amino acids & derivatives**						
Peptides	7755/7990	4 (13)	6 (24)	12 (37)	8 (29)	5 (18)
	25906/16670					
Alpha amino acids	2642	6 (6)	3 (3)	1 (1)	2 (2)	2 (2)
Putrescine	8650	1 (1)				
D-nopaline	7620		1 (4)			
Psicosyl-lysine	61425		1 (1)			
Fructosyl-lysine	61393		1 (1)			
L-cysteine	6207			1 (1)		
**Sugars & derivatives**						
Beta-glucosides	60980/3522		1 (5)	1 (4)	2 (2)	
Nucleosides	7647/23636		1 (6)	2 (8)		
Glucose	5418			1 (4)	2 (5)	
Fructosyl-lysine	61393		1 (1)			
Glycerates	24347		1 (1)			
Psicosyl-lysine	61425		1 (1)			
2-(E)-O-feruloyo-D-galactarate(2-)	58901		1 (1)			
Maltose	6668			1 (4)		
Sucrose	9314			1 (4)		
Fructose	5172			1 (4)		
Melibiose	6733				2 (2)	
Mannose	14575				1 (4)	
UDP-sugars	9840					7 (7)
Pentosides	35312					2 (2)
**Drugs/antibiotics**						
Antimicrobial agents	33281	1 (1)	10 (13)	7 (7)	6 (6)	9 (11)
Erythromycin A	4841		1 (1)	3 (3)	5 (5)	7 (8)
Macrolides	25106		2 (2)	3 (3)	5 (5)	9 (11)
3,6-diamine-10-methylacridinium	383703		3 (3)	5 (5)	3 (3)	1 (1)
(E)-4-(trimethylammonium)but-2-enoate	17237		2 (2)	1 (1)	3 (3)	
Oleandomycin	7737			1 (1)	1 (1)	3 (4)
Azithromycin	2955			1 (1)	1 (1)	2 (2)
Alphaprodine	135075		1 (2)			1 (2)
Quinolone	23765		1 (1)			1 (1)
Tetraphenylphosphonium	9502			1 (1)		1 (1)
Verapamil	9948			1 (1)		1 (1)
Vincaleukoblastine	9983			1 (1)		1 (1)
Ethidium	42478			1 (1)		5 (5)
2’-(4ethoxyphenyl)-5-(4-methylpiperazin-1-yl)-2,5’-bibenzimidazole	5742			1 (1)		2 (3)
Lantibiotics	71644			1 (1)		
Fludarabine	63599				1 (1)	
Colistin	659853				1 (1)	
Thiazole	30637				1 (1)	
Beta-lactam antibiotics	10427					1 (2)
Norfloxicin	100246					4 (4)
**Anions**						
Electrons	10545	1 (1)	1 (1)	5 (13)	1 (1)	2 (2)
Fatty anions	4984	1 (1)	7 (7)	5 (5)	22 (22)	14 (14)
Phosphate	7793	3 (9)	4 (7)	4 (4)	7 (7)	5 (5)
Arsenite	2846	3 (4)		1 (1)	1 (1)	1 (1)
Selenite	9090	1 (1)			2 (2)	1 (1)
Nitrite	7585		1 (3)	1 (2)		1 (3)
Antimonite	30297	2 (2)		1 (1)		
Vanadate	9929	1 (3)			1 (3)	
Cyanate	23419		1 (3)			1 (3)
Fluoride	17051	1 (1)				
Nitrate	7580			1 (2)		
Tungstate	46502			1 (3)		
Tellurite	30477				1 (1)	
Sulfite	9344					1 (1)
**Cations**						
Iron (Fe^2+^)	34754	1 (1)	4 (5)	10 (10)	5 (5)	3 (6)
Sodium (Na^+^)	9175	2 (10)	6 (14)	5 (14)	4 (4)	6 (6)
Copper (Cu^2+^)	23380/49551	1 (1)	1 (1)	5 (5)	6 (6)	2 (2)
Vitamins	33229/5140	1 (4)	3 (6)	7 (7)	3 (6)	2 (6)
	9530/8843					
	7559/17015					
Zinc (Zn^2+^)	10113/49972	2 (4)	1 (1)	4 (4)	5 (5)	3 (5)
Calcium (Ca^2+^)	3308	2 (2)	3 (3)	4 (4)	7 (7)	6 (6)
Protons (H^+^)	5584/24636	8 (40)	13 (37)	11 (35)	11 (23)	5 (11)
Magnesium (Mg^2+^)	6635	2 (2)	1 (1)	1 (1)		1 (1)
Nickel (Ni^2+^)	25517	1 (4)		1 (1)	1 (1)	1 (3)
Cobalt (Co^2+^)	23337	3 (6)		3 (3)		2 (4)
Manganese (Mn^2+^)	29035		1 (3)		2 (2)	3 (5)
Potassium (K^+^)	8345			1 (1)	1 (1)	4 (4)
Choline/osmolytes	3665/25728		1 (1)	4 (4)		
Silver (Ag^+^)	49468				2 (2)	1 (1)
**Macromolecules**						
Proteins	8526/14911	4 (9)	2 (7)	4 (11)	4 (9)	2 (7)
Lipid precursors	25051/18059	3 (3)	8 (8)	7 (7)	22 (22)	15 (15)
Polysaccharides (glycosyl residues)	24403	1 (1)	2 (2)	7 (7)	3 (3)	
siderophores	26672/31432	1 (1)	2 (2)	1 (1)		1 (1)
Diterpenes	35190		1 (1)			1 (1)
Exo-polysaccharides	72813	1 (2)				
Lipopolysaccharide	6494	1 (1)				
Beta-1,4-glucans	3529			1 (1)		
**Systems without inferred substrates:**		48 (56)	112 (138)	170 (244)	145 (169)	202 (237)

The table provides the number of TC systems and, in parenthesis, the number of proteins involved in the transport of different substrates types.

### Transport proteins encoded within the fully sequenced genome of *Candidatus Prometheoarchaeum syntrophicum* MK-D1

After completion of the analyses of transportomes for the 4 MAGs, a publication describing the cultivation of a Lokiarchaeon, which the authors named *Candidatus* Prometheoarchaeum syntrophicum MK-D1 (see last paragraph of the Introductory section; this organism is abbreviated MK-D1) [38]. Hiroyuki Imachi with 21 co-authors spent over 10 years culturing this archaeon, which had a final generation time of about 20 days and was dependent on the presence of a “helper” bacterium. The genome sequence was published allowing us to analyze the transport proteins encoded within it [38]. The data are presented in the last columns of Tables 1–3. We briefly summarize the results in this section. Table 2 shows that in general, those TC subclasses best represented in the four MAGs are also well represented in the MK-D1 genome (last column). It is evident that the numbers of secondary active carriers (subclass 2.A) in both the MAGs and MK-D1 are more prevalent than the primary active transporters (subclass 3.A), and the only other category with as many systems were in the unknowns of TC subclass 9.B. Table 2 also shows that other subclasses were represented in all proteomes: 1.A (alpha-type channel proteins), 3.D (Oxidoreduction-driven transporters), 4.C (Acyl CoA ligase-coupled putative transporters), 4.D (Polysaccharide synthase/exporters), 4.F (Choline/Ethanolamine Phosphotransferases), 8.A (Auxiliary transport proteins), and 9.A (Transporters of unknown biochemical mechanisms. All other TC subclasses were not fully represented. For example, members of subclass 1.C were only identified in Loki and MK-D1. In the proteomes of Thor and Heimdall, holin-like proteins were found (TC# 1.E.43), but none was found encoded within the Odin or Loki MAGs and the MK-D1genome. Systems in subclass 5.A (Transmembrane 2-electron transfer carriers) were identified in all proteomes, except for that of Odin. Thus, many different types of transporters and group translocators were found in the MAGs and the MK-D1 genome. Of particular note is that not a single outer membrane pore-forming protein (porin) was found either in the MAGs or in the fully sequenced MK-D1 genome, as expected for a group of monoderm (single membrane) organisms [163]. Moreover, not a single protein of the phosphoenolpyruvate-dependent sugar-transporting phosphotransferase system (TC subclass 4.A), found in many prokaryotes, was identified although these systems are common in strict and facultative anaerobes of both archaea and bacteria [164–166].

Although there was a general correlation between the proteins encoded within the MK-D1 genome and the MAGs as noted above, one protein in particular was found in the former that was not detected in the latter. This was a homolog of the recently discovered Heliorhodopsin Family (TC# 3.E.3) [167]. Many organisms capture or sense sunlight using rhodopsin pigments, integral 7 transmembrane spanning segment (7 TMS) membrane proteins that bind retinal chromophores. Rhodopsin, both from animals and microorganisms, are members of the TOG superfamily [168]. The 7 helices form a pocket in which retinal is linked covalently via a protonated Schiff base to a lysine residue in the seventh TMS [167]. Heliorhodopsins are distantly related to microbial rhodopsins (TC# 3.E.1), and animal rhodopsins (TC# 9.A. 14.1), but they are embedded in the membrane with their N termini facing the cell cytoplasm, an orientation that is opposite to that of microbial and animal rhodopsins. Heliorhodopsins show photocycles that last about 5 seconds, suggesting that they have light-sensory activities. The photocycles accompany retinal isomerization and proton transfer, as in other rhodopsins. However, in contrast to these well-characterized rhodopsins, protons are not believed to be released from the heliorhodopsin protein, even transiently. Thus, they apparently do not transport protons across the membrane. Heliorhodopsins are abundant and distributed globally in Archaea, Bacteria, Eukarya and their viruses [169], and are thus widespread in the microbial world [167]. The presence of such a protein in MK-D1 means that these proteins may in general be used by members of the Asgard superphylum for the detection, and therefore, utilization (in some unknown way) of solar energy.

Table 3 summarized the substrates probably transported by MK-D1 (last column) based on the substrates annotated in TCDB for TC systems identified in this genome as described in Methods. The substrates fall into several categories as indicated in Table 3. This organism takes up organic nutrients including amino acids, peptides and their derivatives as sources of carbon, energy and nitrogen as well as precursors for protein synthesis. It also takes up various sugars including hexoses, pentoses and their glycosides as well as sugar acids and other sugar derivatives. It takes up metabolites including organic acids, especially fatty acids, all of which can serve as sources of carbon and energy. The organism also transports a variety of inorganic cations, some of them essential for life (e.g., H^+^, Na^+^, K^+^, Mg^2+^, Fe^2+^ and other heavy metal ions) which are taken up into the cells and/or are extruded. It is particularly interesting to note that these organisms have transporters for reduced iron (Fe^2+^) but apparently not oxidized iron (Fe^3+^). This fact is in accordance with expectation since these organisms were isolated from anaerobic environments where Fe^2+^, but not Fe^3+^ should predominate. Asgard archaea also transport some toxic cations, (e.g., Ag^+^, Cd2^+^, Cu2^+^) which are exported from the cells. Similarly, many inorganic anions, some of them providing essential elements for life (e.g., nitrate. nitrite, phosphate, sulfate, sulfite, selenite) are taken up, while toxic anions (e.g., antimonite, arsenite, fluoride) are extruded. Electrons are included as anionic species in Table 3 only if they are transported from one side of the membrane (e.g., the cytoplasm) to the other (e.g., the external environment). In the analyses summarized in Table 3, the primary substrates transported, and not the co-transported cations (H^+^ or Na^+^) which function only to provide energy for the uptake or extrusion of other compounds, are included. It can be seen that, except for MK-D1, there are more H^+^ transporters than for any other inorganic cation. These are transported by cation (Na^+^ or K^+^):H^+^ anti-porters as well as by proton-pumping ATPases, pyrophosphatases and electron transfer complexes.

The largest class of compounds transported by MK-D1 and the four Asgard MAGs are hydrophobic and amphipathic compounds such as drugs, antibiotics and secondary metabolites that are excreted from the cytoplasm into the external environment (Table 3). These are in general exported via multidrug resistance (MDR) pumps, both primary and secondary active transporters, with the latter predominating. An examination of Table 3 reveals the variety of antibiotics and other drugs probably secreted against large concentration gradients by these organisms. Protection from noxious substances is clearly important for the Asgard archaea, but it should be kept in mind that the export of biosynthesized secondary metabolites by Asgard such as endogenous small molecular toxins, biosynthetic metabolites (dyes, siderophores, chelating agents, etc.) and end products of metabolism are often the physiologically relevant substrates of these systems. Nevertheless, it seems clear that a fair amount of energy is expended for cell protection against noxious compounds, either those synthesized by themselves, or those produced by other organisms. While exopolysaccharides are clearly synthesized and exported by Asgard archaea, no such exporter was identified encoded within the MK-D1genome. However, all transportomes include protein exporters as well as some that transport diterpenes and other lipid-like molecules as well as a few possible siderophores, which may, however, not be the true substrates of these transporters, as siderophores function to chelate Fe^3+^ which is not likely to predominate in an anaerobic environment, and no iron-siderophore uptake transporters were identified. It is worth noting, however, that in all proteomes there is a large number of (putative) transporters (Table 2) and systems without inferred substrates (Table 3).

## Conclusions

The Asgard superphylum is hypothesized to be the closest known archaeal relative to the first eukaryote [3, 4, 33]. The Asgard MAGs and MK-D1 encode eukaryotic signature protein (ESP) homologs involved in cytoskeleton remodeling and primitive ER-golgi vesicular trafficking. Based on the publicly available MAG data from the four phyla of the Asgard (Odin, Thor, Heimdall, and Loki), the consensus of research has concluded that most metabolism in the Asgard superphylum is mixotrophic and anaerobic.

All Asgard proteomes include a diverse range of transport systems capable of generating a *pmf*. The *pmf* thus generated can drive oxidative phosphorylation via the proton pump complexes present universally in the Asgard. Alternatively, the *pmf* could be utilized to support electrochemical potential-driven transport by secondary carriers. The latter is more likely due to the greater percentage of secondary carriers than ATP-driven transporters within these Asgard organisms. To a lesser extent, the sodium membrane potential is likely to be generated in all Asgard metagenomes.

Based on the analysis of the transportome encoded within these four Asgard MAGs and the genome of MK-D1, we conclude that organisms in the Asgard superphylum are metabolically versatile with multiple means of acquiring energy for growth. Loki and Thor likely take up a diverse range of organic compounds such as lipid precursors, sugars, amino acids, and proteins. However, while Thor’s intake of organic molecules is more balanced, Loki and MK-D1 contain about twice the number of lipid precursor transporters. Additionally, both Loki and Thor seem to be capable of anaerobic respiration, and they contain some components necessary for methanogenesis. Odin and Heimdall take up organic molecules, mostly amino acids, amino acid derivatives and peptides. Odin may have the most diverse metabolic repertoire of all the Asgard organismal types examined and contains the most components of transport systems required for methanogenesis. Due to the presence of systems that utilize oxygen and fumarate as electron acceptors, Heimdall may be capable of respiration in both anaerobic and aerobic environments.

The primary type of transport systems present across the Asgard superphylum is the secondary carriers, which indicates Asgard’s reliance on sustained membrane potentials. All Asgard proteomes contain a large percentage of drug exporters, which may confer drug and toxin resistance to Asgard within their respective environments. Further analyses of the transportomes of multiple species within each phylum will be required to determine whether the trends observed in this study are uniquely characteristic of these phyla.

The five Asgard transportomes contain a limited number of eukaryotic type systems. Nevertheless, the transporters encoded within these Asgard MAGs were either ubiquitous to all domains of life, or prokaryotic in nature; a relatively few transporters typical of eukaryotes were identified. These homologs include Mg^2+^-specific ion channels and eukaryotic-like orthologs of glycosyl transferases in all Asgard metagenomes. Furthermore, multiple systems in Heimdall were homologous to uncharacterized ABC transporters unique to eukaryotes. Odin and Heimdall contain proteins homologous to eukaryotic presenilins. In addition, Loki contains a putative eukaryotic-like V-type ATP synthase. Thus, based on the analysis of transport systems, these findings are in agreement with claims regarding Asgard’s close phylogenomic relationship to eukaryotes.

Contingent upon the veracity of Asgard’s proposed ancestral relationship to eukaryotes, these findings suggest that the last eukaryotic common ancestor lacked many of the transporters commonly found in modern eukaryotes and, thus, the complexity of the eukaryotic transportome may have developed after the primary endosymbiotic event that is claimed to involve the Asgard host cell and a proteobacterium that gave rise to mitochondria. However, it is important to note that limited metagenomic data as well as one completely sequenced Loki genome were available for this study. Therefore, it is questionable whether the scarcity of eukaryotic transport components has a direct bearing on the question of the origin of the eukaryotic cell. While more work is needed to determine if the unique traits of these four Asgard transportomes are characteristic of each phylum, it is essential that the genomic data for all Asgard organisms be expanded.

## Supporting information

S1 Table Conservation of transport proteins and their substrates across five Asgard proteomes. The first 2 columns show the TC IDs (TCIDs) and protein accessions (Accessions) of the homologs in TCDB that were used to annotate the candidate transporters identified in the Asgard proteomes. The third and fourth columns describe the substrates transported by the TC protein based on the ChEBI ontology [170] accessions for chemical entity (ChEBI_chemical_entity) and biological role (ChEBI_role), respectively. The fifth column (TC_hit_tms_number) shows the number of TMSs inferred by HMMTOP [46] in the TC protein. Columns 6–10 show the presence (+) or absence (-) of a given TC system in the proteomes of Odin, Thor, Heimdall, Loki and MK-D1. Columns 11–16, 17–22, 23–28, 29–34 and 35–40 contain information describing the alignments between TC proteins (i.e., the hits) and the top matches from Asgard candidate transporters (i.e., the queries) in Odin, Thor, Heimdall, Loki and MK-D1, respectively. These 6-column blocks show for each proteome the accession of the corresponding Asgard protein (query), the number of HMMTOP-predicted TMSs (q_tms) in the Asgard protein, the Smith-Waterman E-value (evalue) of the alignment as implemented in SSEARCH [171], the percent identity (pident), and the alignment coverages for both the Asgard (qcov) and TC proteins (scov).
(XLSX)
